# Inhibition of stearoyl-CoA desaturase 1 (SCD1) enhances the antitumor T cell response through regulating β-catenin signaling in cancer cells and ER stress in T cells and synergizes with anti-PD-1 antibody

**DOI:** 10.1136/jitc-2022-004616

**Published:** 2022-07-06

**Authors:** Yuki Katoh, Tomonori Yaguchi, Akiko Kubo, Takashi Iwata, Kenji Morii, Daiki Kato, Shigeki Ohta, Ryosuke Satomi, Yasuhiro Yamamoto, Yoshitaka Oyamada, Kota Ouchi, Shin Takahashi, Chikashi Ishioka, Ryo Matoba, Makoto Suematsu, Yutaka Kawakami

**Affiliations:** 1 Division of Cellular Signaling, Institute for Advanced Medical Research, Keio University School of Medicine, Tokyo, Japan; 2 Division of Anatomical Science, Department of Functional Morphology, Nihon University School of Medicine, Tokyo, Japan; 3 Department of Biochemistry, Keio University School of Medicine, Tokyo, Japan; 4 Department of Obstetrics and Gynecology, Keio University School of Medicine, Tokyo, Japan; 5 Laboratory of Veterinary Surgery, Graduate School of Agricultural and Life Sciences, The University of Tokyo, Tokyo, Japan; 6 National Hospital Organisation Tokyo Medical Center, Tokyo, Japan; 7 Department of Respiratory Medicine, The University of Tokyo, Tokyo, Japan; 8 Department of Medical Oncology, Tohoku University Hospital, Sendai, Japan; 9 Department of Clinical Oncology, Tohoku University Graduate School of Medicine, Sendai, Japan; 10 DNA Chip Research Inc, Tokyo, Japan; 11 Department of Immunology, International University of Health and Welfare, Chiba, Japan

**Keywords:** Immunotherapy, Tumor Microenvironment, CD8-Positive T-Lymphocytes, Drug Therapy, Combination

## Abstract

**Background:**

Understanding the mechanisms of non-T cell inflamed tumor microenvironment (TME) and their modulation are important to improve cancer immunotherapies such as immune checkpoint inhibitors. The involvement of various immunometabolisms has recently been indicated in the formation of immunosuppressive TME. In this study, we investigated the immunological roles of stearoyl-CoA desaturase 1 (SCD1), which is essential for fatty acid metabolism, in the cancer immune response.

**Methods:**

We investigated the roles of SCD1 by inhibition with the chemical inhibitor or genetic manipulation in antitumor T cell responses and the therapeutic effect of anti-programmed cell death protein 1 (anti-PD-1) antibody using various mouse tumor models, and their cellular and molecular mechanisms. The roles of SCD1 in human cancers were also investigated by gene expression analyses of colon cancer tissues and by evaluating the related free fatty acids in sera obtained from patients with non-small cell lung cancer who were treated with anti-PD-1 antibody.

**Results:**

Systemic administration of a SCD1 inhibitor in mouse tumor models enhanced production of CCL4 by cancer cells through reduction of Wnt/β-catenin signaling and by CD8^+^ effector T cells through reduction of endoplasmic reticulum stress. It in turn promoted recruitment of dendritic cells (DCs) into the tumors and enhanced the subsequent induction and tumor accumulation of antitumor CD8^+^ T cells. SCD1 inhibitor was also found to directly stimulate DCs and CD8^+^ T cells. Administration of SCD1 inhibitor or SCD1 knockout in mice synergized with an anti-PD-1 antibody for its antitumor effects in mouse tumor models. High SCD1 expression was observed in one of the non-T cell-inflamed subtypes in human colon cancer, and serum SCD1 related fatty acids were correlated with response rates and prognosis of patients with non-small lung cancer following anti-PD-1 antibody treatment.

**Conclusions:**

SCD1 expressed in cancer cells and immune cells causes immunoresistant conditions, and its inhibition augments antitumor T cells and therapeutic effects of anti-PD-1 antibody. Therefore, SCD1 is an attractive target for the development of new diagnostic and therapeutic strategies to improve current cancer immunotherapies including immune checkpoint inhibitors.

**WHAT IS ALREADY KNOWN ON THIS TOPIC**

- Although involvement of lipid metabolism in cancer immunity was suggested, the role of fatty acid desaturase stearoyl-CoA desaturase 1 (SCD1) in cancer immune responses has not been investigated. In this study, we attempted to clarify the mechanisms of SCD1 in regulating immune response to cancer cells.

**WHAT THIS STUDY ADDS**

- Inhibition of SCD1 in cancer cells via suppressing β-catenin signaling and in effector T cells via reducing endoplasmic reticulum stress enhances antitumor T cells through CCL4 recruited dendritic cells and synergizes with antiprogrammed cell death protein 1 (anti-PD-1) antibody. In human cancers, SCD1 is highly expressed in one of the non-T cell inflamed subtypes of colon cancer, and serum SCD1 related free fatty acids are correlated to the responses to anti-PD-1 antibody therapy in patients with non-small cell lung cancer, indicating immunosuppressive functions of SCD1 in patients with cancer.

**HOW THIS STUDY MIGHT AFFECT RESEARCH, PRACTICE AND/OR POLICY**

- SCD1 and the related free fatty acids were found to be attractive targets for diagnosis and therapy to improve current PD-1/PD-L1 inhibitor-based combination immunotherapy.

## Introduction

Cancer immunotherapies, including immune checkpoint inhibitors (ICIs) (eg, with antibodies to programmed cell death protein 1 (PD-1)/programmed cell death 1 ligand 1 (PD-L1) and cytotoxic T-lymphocyte-associated protein 4) have shown durable clinical effects in patients with various types of cancers.^1–3^ However, their efficacy remains limited to a subset of patients.^4^ Analysis of pretreatment tumor biopsy samples from patients treated with checkpoint blockade therapy revealed that patients with pre-existing local antigen-specific CD8^+^ T cell infiltration (T cell inflamed) that indicates potential induction of T cells for relatively high immunogenic cancers were more likely to show a clinical response.^4–6^ However, most solid tumors have an immunosuppressive tumor microenvironment (TME) without T cell infiltration (non-T cell inflamed), in which cases treatment with anti-PD-1 antibodies alone are ineffective.^4^ It is therefore necessary to identify biomarkers to predict clinical effects and therapeutic targets for the development of effective combination therapies by improving the immunosuppressive TME in refractory cases.^4 7^

Stearoyl-CoA desaturase 1 (SCD1) is the rate-limiting enzyme involved in the synthesis of monounsaturated fatty acids (MUFAs) such as palmitoleic acid (16:1 n-7) and oleic acid (18:1 n-9), from saturated fatty acids such as palmitic acid (16:0) and stearic acid (18:0).^8 9^ SCD1 is considered as a potential therapeutic target for cancers because it is expressed at high levels in multiple types of cancers and plays an important role in cancer cell growth^9–11^ by controlling fatty acid metabolism, which is necessary to various cellular functions including maintaining cell membrane components, regulating endoplasmic reticulum (ER) stress signals^12–15^ and oncogenic signals such as the Wnt/β-catenin pathway.^16 17^

Recently, these SCD1-related signals, such as the Wnt/β-catenin pathway and ER stress signals, have been reported to play an important role in antitumor immune responses. Activation of the β-catenin pathway has been reported to be related to non-T cell inflamed tumors in various types of human cancers including melanoma, colorectal cancer and liver cancer. Reductions of DC recruiting chemokines, such as C-C motif chemokine ligand 4 (CCL4),^18^ or increases of immunosuppressive cytokines such as IL-10,^19^ from β-catenin-activated cancer cells was reported as its immunosuppressive mechanism. ER stress signals have also been reported to exert immunomodulatory effects in the TME. For example, nutrient restriction and reactive oxygen species accumulation in the TME cause ER stress in intratumoral T cells, which leads to mitochondrial dysfunction and inhibition of their anticancer effector function.^20–22^

Although previous studies have investigated the role of SCD1 in the characteristics of cancer cells, the involvement of SCD1 in regulating immune cells, immune cell functions and in antitumor immune responses has not yet been elucidated. In this study, we evaluated the roles of SCD1 in an immunosuppressive TME and antitumor T cell responses and demonstrate that SCD1 and related fatty acids both in cancer cells and in immune cells are attractive targets as biomarkers and therapeutic targets for ICI-based combination immunotherapy.

## Materials and methods

### Animals and cell culture

Mice were bred at the animal facilities of Keio University, in accordance with the guidelines for animal experimentation. Mice were maintained in a specific pathogen-free environment on a 12-hour light–dark cycle, with the dark cycle occurring from 20:00 to 08:00. SCD1-global deficient mice with a C57BL/6 J background (B6.129-Scd1tm1Ntam/J;006201) were purchased from Jackson Laboratory. The murine colorectal carcinoma cell line (CT26) and murine mammary adenocarcinoma cell line (4T1), murine T cell lymphoma cell line (EL4) and human colon cancer cell line (HT29) were purchased from the American Type Culture Collection. The murine colon adenocarcinoma cell line (MC38) and murine sarcoma cell line (MCA205) and the human melanoma cell lines, 1861mel and 938mel, were obtained from the Surgery Branch of the National Cancer Institute, National Institutes of Health. Human T cells were cultured in AIM-V medium (Thermo Fisher Scientific) containing 10% heat-inactivated human AB serum and 300 IU/mL recombinant human interleukin-2 (Novartis). Mouse T cells and human and mouse cancer cells were cultured in RPMI 1640 (Thermo Fisher Scientific) containing 10% heat-inactivated fetal bovine serum (FBS), 100 U/mL penicillin, and 100 µg/mL streptomycin. The concentration of FBS in the SCD1 inhibition experiments was 2%.

## Patients

The current study enrolled 57 patients diagnosed with stage II, III and IV colorectal cancer at the Tohoku University Hospital (Sendai, Japan) and 24 patients diagnosed with NSCLC at the Tokyo Medical Center (Tokyo Japan). PFS and OS were calculated using the Kaplan-Meier method with the use of a log-rank test. PFS was defined as the time from the start of treatment to documented evidence of progressive disease or death. OS was defined as time from treatment initiation to death from any cause. The cut-off used the median. A responder was defined as a patient with ‘complete response’ or ‘partial response’ as determined by RECIST V.1.1 criteria. All participants provided written informed consent before participation in the study.

### Tumor-bearing mouse models

C57BL/6, Balb/c and SCD1 KO mice aged 6–8 weeks were inoculated subcutaneously in the flank with 5×10^5^ MC38, 5×10^5^ CT26, 7×10^5^ MCA205, 7×10^5^ 4T1 cells or 5×10^5^ MC38-DsRed cells on day 0. On day 4, mice were treated with vehicle (50% v/v polyethylene glycol 400, 20% v/v propylene glycol, 20% v/v vitamin E, 5% v/v ethanol, 5% w/v polyvinylpyrrolidone) or with 10 mg/kg SCD1 inhibitor (A939572) in vehicle. A939572 was purchased from APExBIO. Mice received the vehicle or the inhibitor in vehicle via oral gavage twice daily for 16 days. Mice in the combined therapy group also received anti-PD-1 (J43) or isotype-matched antibody (200 µg/body; Bio X Cell,) on days 4, 7 and 10. For cell depletion, anti-CD8 or isotype antibody (200 µg/body; Bio X Cell) was given intraperitoneally on days 1, 4, 5, 6 and 10. Tumor volume was calculated by direct tumor measurements every 4 days, using the formula: [length × (width)^2^]/2.

### Samples and sample preparation for gas chromatography-mass spectrometry (GC-MS)

To 20 µL of serum, 0.3 mL of PBS containing an internal standard (100 ng of margaric acid) was added and mix with vortex mixer. Draining lymph nodes cells (1×10^6^ cells) and mouse cancer cell lines (1×10^5^ cells) were sonicated with 0.3 mL of PBS containing an internal standard. Free fatty acids were extracted using ISOLUTE SLE +column and dichloromethane. The organic fractions were dried under nitrogen stream. Tumor tissue sample were grounded using a multibead shocker (MB755U, Yasui Kikai) with a frozen disruption device, and 1 mL methanol containing an internal standard was added. After addition of 0.5 mL of deionized water and 0.8 mL chloroform, the mixture was centrifuged at 20 000× g for 15 min at 4°C. The lower organic layer of the sample was collected and dried under nitrogen stream. The residue was dissolved in 5 µL of pyridine and 30 µL of the reagent BSTFA+TMCS (99:1) (TS-38831, Thermo Fisher Scientific) for trimethylsilylation. The derivatisation reaction was performed for 30 min at 40°C.

### GC-MS analyses

GC-MS analysis was performed on a Shimadzu GC-MS QP2010 Ultra equipped with an AOC20i autoinjector and Rtx-5MS column (30 m, 0.25 mm, 0.25 µm df) in the 70 eV electron ionization mode. The oven temperature program was as follows: 150°C for 1 min, 20 °C/min to 250°C, 5 °C/min to 280°C, hold 5 min. then 20 °C/min to 330°C, hold for 3 min where the temperature was maintained for 10 min. The carrier gas was helium with a constant flow speed of 42.0 cm/sec. One microliter was injected in 5:1 split ratio with an injector temperature of 250°C, MS interface temperature was held at 280°C. Selected ion monitoring for quantification was performed by recording the ions at *m/z* 311.20 for palmitoleic acid-trimethylsilyl derivative, *m/z* 313.20 for palmitic acid-trimethylsilyl derivative *m/z* 327.20 for margaric acid-trimethylsilyl derivative, *m/z* 339.20 for oleic acid-trimethylsilyl derivative, and *m/z* 341.20 for stearic acid-trimethylsilyl derivative, respectively.

## Results

### The SCD1 inhibition enhances antitumor T cells through recruiting DCs into tumors

**Figure 1:**
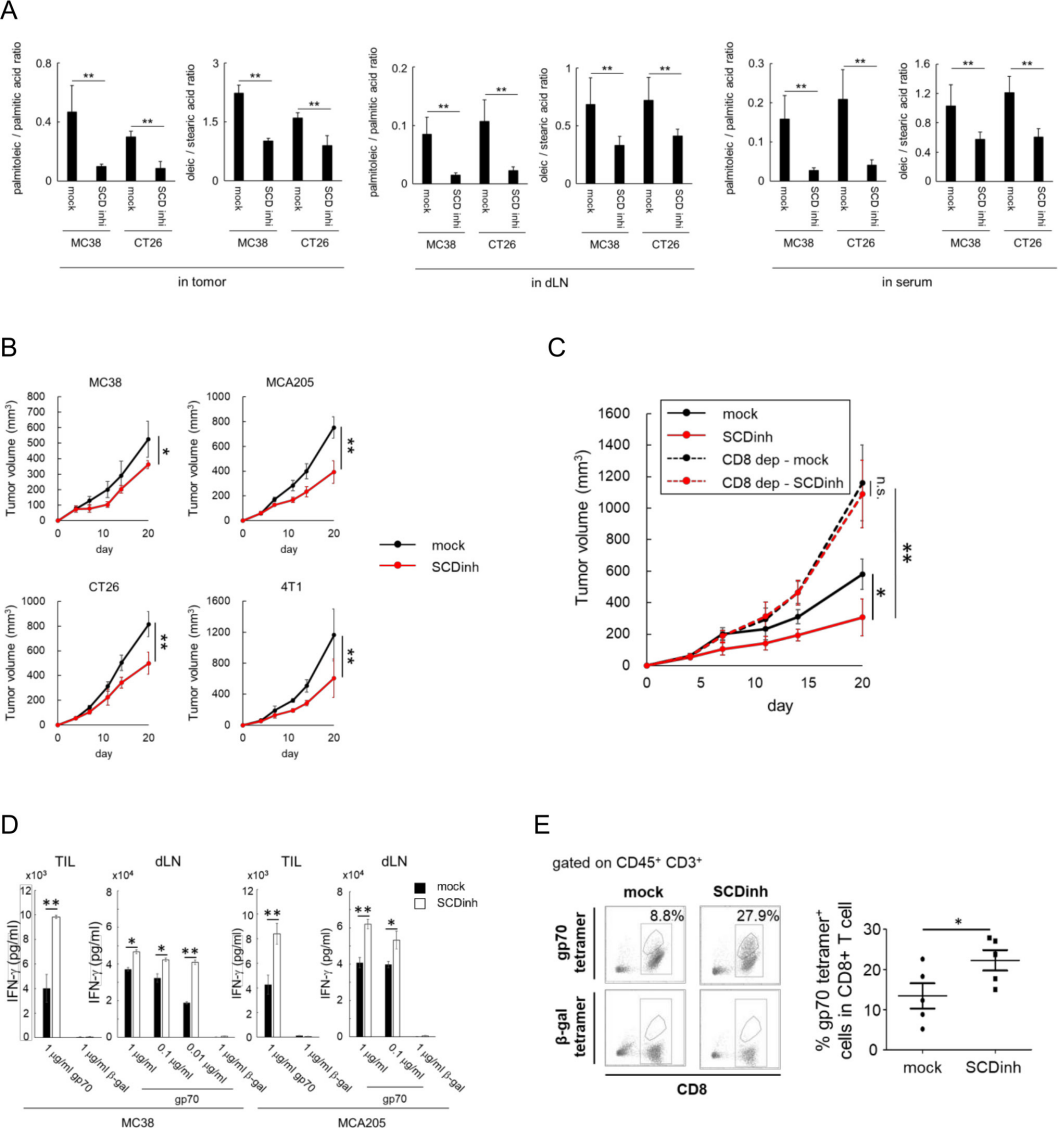
Inhibition of SCD1 enhances antitumor immune responses. C57BL/6 mice bearing MC38 or MCA205 tumors and Balb/c mice bearing CT26 or 4T1 tumors were treated with a SCD inhibitor (SCDinh) or with vehicle only (mock). (A) Ratios of palmitoleic acid / palmitic acid and oleic acid / stearic acid in the tumor, draining lymph nodes (dLN) and sera in C57BL/6-MC38 and Balb/c-CT26 model. (B) Tumor-growth curves in mean tumor volumes (mm3 ± standard deviation (SD); n=5) in four models. (C) Mean tumor volumes (mm3 ± SD; n=5) in MC38 tumor-bearing C57BL/6 mice that received a CD8-depleting or an isotype-matched monoclonal antibody. (D) Tumor‐infiltrating CD8+ T cells and irradiated syngeneic splenocytes cocultured and restimulated with gp70 peptide or β-gal peptide (negative control). In vivo tumor antigen‐specific T‐cell induction from tumor (TIL) and dLN evaluated by IFN-γ release assays in C57BL/6-MC38 (left panel) and in C57BL/6-MCA205 (right panel) models (means ± SD; n=3). (E) Percentages of gp70-specific CD8+ T cells in Balb/c-CT26 tumors analyzed by flow cytometry. Representative gp70-tetramer staining of CD8+ T cells in each group (left panel) and for all individuals (right panel) (n=5). *P<0.05, **P<0.01. Dep, depleted.

In order to clarify the role of fatty acid metabolic enzyme SCD1 in the cancer-related immune response, we evaluated the immunologic antitumor effects of SCD1 inhibition and its mechanism using C57BL/6 and Balb/C mice implanted with four different types of syngeneic murine tumors, including MC38 colon cancer cells, CT26 colon cancer cells, MCA205 sarcoma cells and 4T1 breast cancer cells. SCD1 gene expression, enzymatic activity and direct biological effects of the SCD1 inhibitor in all these cell lines are shown in online supplemental figure 1A–C. Oral administration of the SCD1 inhibitor A939572^23^ inhibited fatty acid desaturation, as shown by the decreased ratios of palmitoleic acid/palmitic acid and oleic acid/stearic acid in the tumor, draining lymph nodes and sera of those tumor-bearing mice (figure 1A) and significantly inhibited the growth of all four tumors (figure 1B).

10.1136/jitc-2022-004616.supp6 Supplementary data

That antitumor effect of inhibiting SCD1 was abrogated by the depletion of CD8^+^ T cells in MC38-bearing mice (figure 1C), indicating the involvement of CD8^+^ T cells in the *in vivo* antitumor effects of the SCD1 inhibitor. SCD1 inhibition significantly enhanced the induction of tumor antigen specific CD8^+^ T cells in tumors and draining lymph nodes in MC38 and MCA 205 bearing mice when evaluated by the interferon (IFN)-γ secreted from tumor antigen gp70-specific T cells (figure 1D) and in CT26-bearing mice when evaluated by gp70/H-2L^d^-tetramer staining (figure 1E).

**Figure 2:**
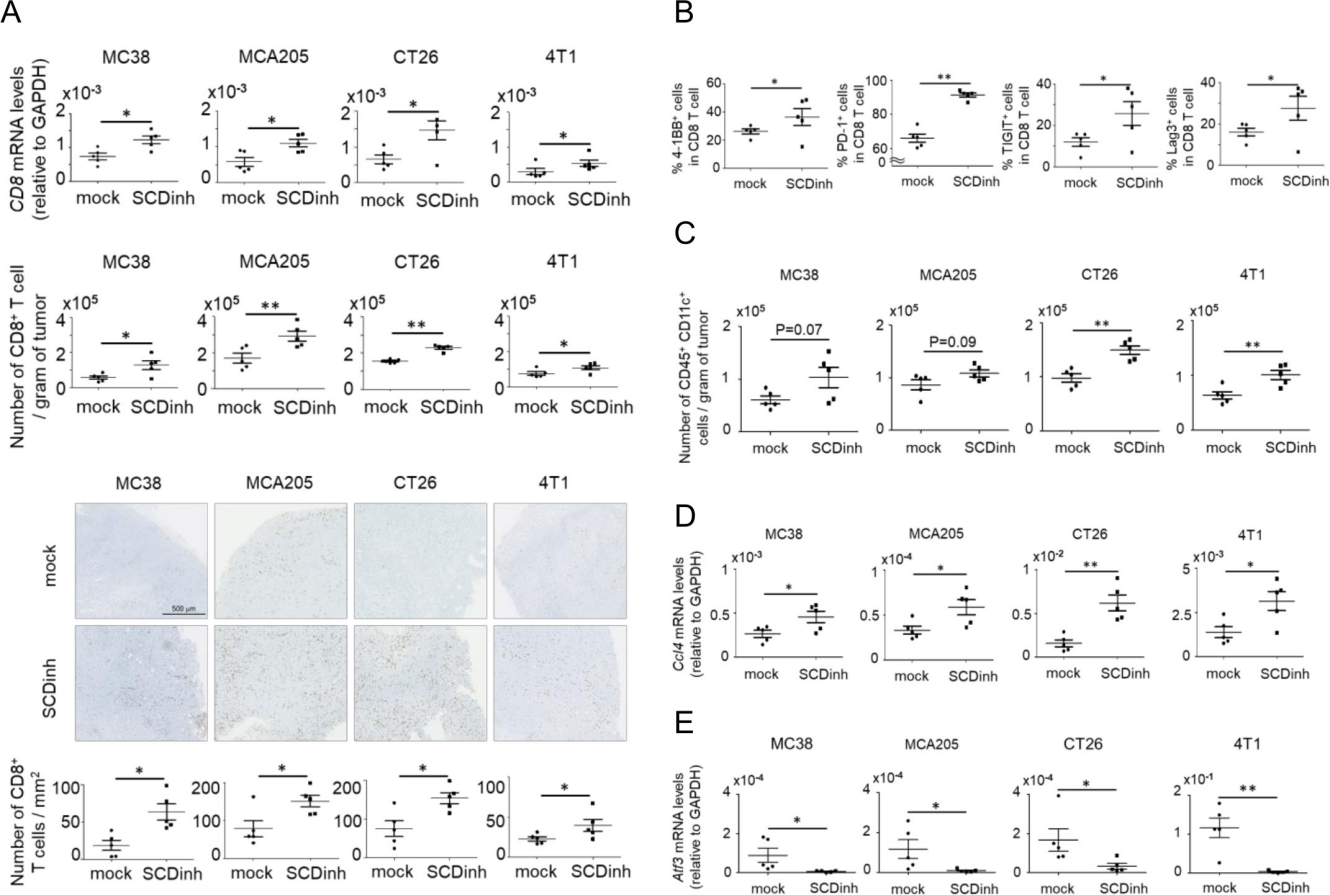
Inhibition of SCD1 enhances the infiltration of CD8+ T cells and DCs into tumors via production of CCL4. C57BL/6 mice bearing MC38 or MCA205 tumors and Balb/c mice bearing CT26 or 4T1 tumors were treated with a SCD inhibitor (SCDinh) or with vehicle only (mock). Tumors were excised on day 20. (A) Analysis of tumor-infiltrating CD8+ T cells by qPCR, flow cytometry, immunostaining and image analysis (n=5). (B) Percentages of 4-1BB+, PD-1+, TIGIT+ and Lag3+ CD8+ T cells in tumors analyzed by flow cytometry in C57BL/6-MC38 model (n=5). (C) Absolute numbers of CD45+ CD11c+ cells analyzed by flow cytometry (n=5). (D, E) Ccl4 and Atf3 gene expression evaluated by real-time RT-PCR (n=5). *P<0.05, **P<0.01. N.D., not determined. Data are expressed as means ± SD.

Administration of the SCD1 inhibitor also increased the infiltration of CD8^+^ T cells into tumors in all four tumor models when analyzed by gene expression analysis by qPCR, flow cytometry and immunohistochemical analyses (figure 2A, online supplemental figure 2 and 3A left panel), although regulatory T cells were not significantly changed (online supplemental figure 3B, C). The expression of stimulatory and inhibitory coreceptors of T cells, including 4-1BB, PD-1, TIGIT and Lag3, was also enhanced in these tumor-infiltrating CD8^+^ T cells by treatment with the SCD1 inhibitor, suggesting the activation and subsequent exhaustion of tumor antigen-specific T cells in tumors (figure 2B, online supplemental figure 3A right panel).

The accumulation of DCs in tumors is known to be important for the induction and effector function of antitumor T cells. In all four tumor models, the tumor-infiltrating CD45^+^ CD11c^+^ DCs were also increased following administration of the SCD1 inhibitor (figure 2C). The expression of CD80, CD83 and CD86 was increased in DCs that infiltrated in CT26 tumors, suggesting the maturation of DCs following SCD1 inhibition (online supplemental figure 3D). Expression of the chemokine CCL4, which is important for recruiting DCs into tumors,^18^ was significantly increased by SCD1 inhibition (figure 2D), accompanied by the decreased expression of activating transcription factor 3 (ATF3) (figure 2E), a transcription factor that inhibits CCL4 expression.^24^ These results suggest that SCD1 inhibition enhances the production of CCL4 in tumor tissues via ATF3 inhibition, which in turn promotes the induction and effector function of tumor antigen specific CD8^+^ T cells through the accumulation and maturation of DCs in tumors.

### The SCD1 inhibition enhances CCL4 production in tumor cells via suppressing Wnt/β-catenin signaling

**Figure 3:**
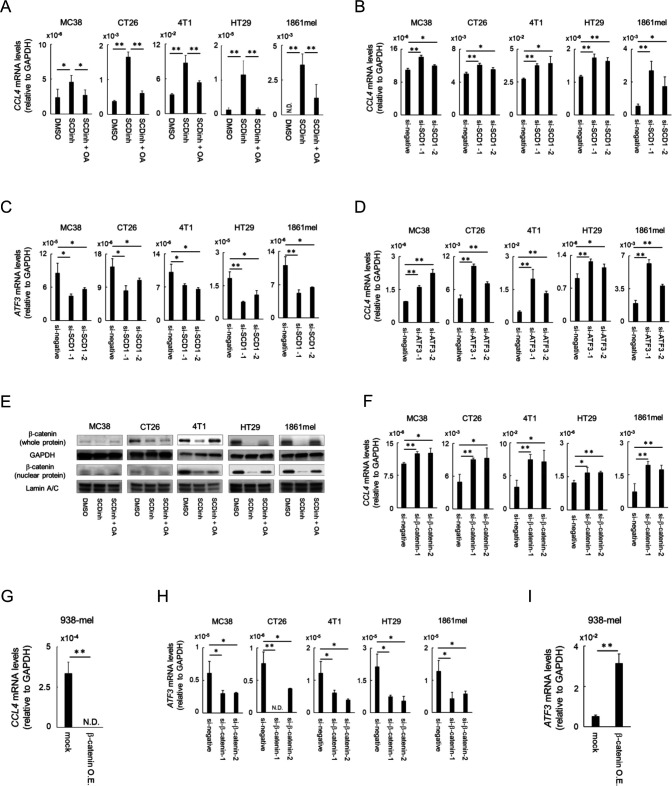
SCD1 regulates CCL4 production via β-catenin in tumor cells. (A) Mouse (MC38, CT26 and 4T1) and human (HT29 and 1861mel) cancer cells were cultured in RPMI medium containing 2% serum supplemented with a SCD inhibitor (SCDinh), with SCDinh + oleic acid (SCDinh + OA) or with dimethylsulfoxide (DMSO). Total RNA was extracted and CCL4 mRNA was evaluated using real-time RT-PCR. (B, C) Cancer cells were transfected with small interfering RNA (siRNA)-SCD1 or with siRNA-control (si-negative) after which CCL4 (B) and ATF3 (C) mRNA levels were evaluated by real-time RT-PCR at 48 h post-transfection. (D) Cancer cells were transfected with siRNA-ATF3 or with siRNA-control after which mRNA levels were evaluated by real-time RT-PCR at 48 h post-transfection. (E) Whole cell and nuclear β-catenin protein levels were measured by western blot; GAPDH and lamin A/C were used as controls. (F-I) Knockdown (F, H) or overexpression (G, I) of β-catenin in mouse and human cancer cell lines. Cancer cells were transfected with siRNA-β-catenin or with si-negative after which CCL4 (F) and ATF3 (H) mRNA levels were evaluated by real-time RT-PCR at 48 h post-transfection. CCL4 (G) and ATF3 (I) gene expression in a human melanoma cell line (938mel) overexpressing β-catenin. Data are expressed as means ± SD (n=3). *P<0.05, **P<0.01. N.D., not determined. O.E., over-expressing.

Tumor cells are reported to be one of the major sources of CCL4 in tumor tissues. We then evaluated production of CCL4 by various tumor cells, including murine tumor cell lines (MC38, CT26, and 4T1), a human cancer cell lines (HT29 colon cancer and 1861mel melanoma) *in vitro*, and attempted to clarify the mechanisms for the inhibition of CCL4 production in tumor cells by SCD1 inhibition. The SCD1 inhibitor significantly enhanced CCL4 gene expression in these tumor cells, which was canceled by the addition of oleic acid, an unsaturated fatty acid produced by SCD1, indicating that oleic acid is involved in the regulation of CCL4 expression in these tumor cells (figure 3A). Knockdown of SCD1 by siRNAs also enhanced CCL4 gene expression (figure 3B) accompanied by reduction of ATF3 expression (figure 3C) and knockdown of ATF3 enhanced the expression of CCL4 (figure 3D) as previously reported.^24^

Since it was reported that SCD1 expression and activity were bidirectionally regulated by the Wnt/β-catenin signaling^16 17^ and that activation of the Wnt/β-catenin signaling inhibited CCL4 production via activation of ATF3 in human melanoma cells,^18^ we evaluated whether SCD1 regulates CCL4 production via β-catenin-ATF3 axis in these tumor cell lines. The SCD1 inhibitor reduced whole and nuclear β-catenin expression (figure 3E), and it was rescued by the addition of oleic acid (figure 3E), suggesting that oleic acid is involved in enhancing β-catenin signaling. Oleic acid was previously reported to stabilize β-catenin levels in cancer cells by preventing its degradation by proteasomes.^25^ Knockdown of β-catenin gene in these tumor cells or overexpression of mutant β-catenin gene in human melanoma cell line 938-mel expressing low β-catenin gene^19^ also regulated SCD1 expression in a negative or positive manner in the tumor cell lines (online supplemental figure 4A, B), indicating that SCD1 and β-catenin regulate their expression in a bidirectional manner. Knockdown (figure 3F) or overexpression (figure 3G) of β-catenin also enhanced or suppressed CCL4 production, respectively, accompanied by the inverse expression of ATF3 (figure 3H, I). These results indicate that bidirectional regulation between SCD1 and β-catenin signaling and downstream ATF3 control CCL4 production in the tumor cells.

**Figure 4:**
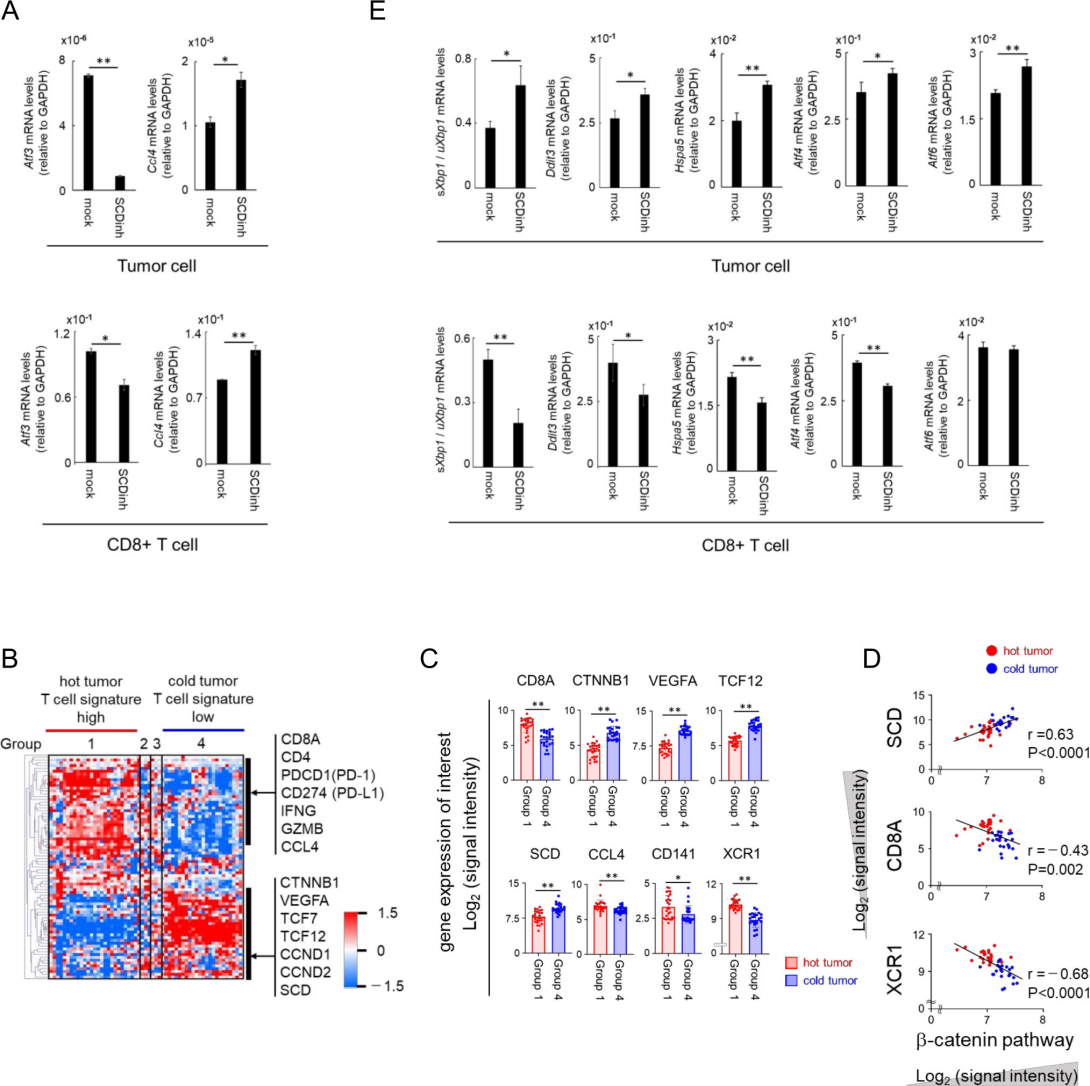
SCD1 is involved in the production of CCL4 and the infiltration of immune cells into tumors via β-catenin and ER stress. (A) C57BL/6 mice bearing DsRed-MC38 tumors were treated with SCDinh or with vehicle after which DsRed+ tumor cells (upper panel) and CD8+ T cells (lower panel) were isolated at day 20. Total RNA was extracted and CCL4 and ATF3 gene expression was analyzed by real-time RT-PCR. (B) Heat map of 57 colorectal tumors classified focusing on immune related genes, fatty acid metabolism related genes and β-catenin pathway genes (dataset 1). (C) Comparison of expression levels of genes of interest (CD8A, β-catenin pathway genes (CTNNB1, VEGFA, TCF12), SCD, CCL4, CD141 and XCR1) in Group 1 (hot tumor; n=27) and in Group 4 (cold tumor; n=24). (D) Correlation analysis of β-catenin pathway (dataset 2) with SCD, CD8A and XCR1 in Group 1 (hot tumor) and in Group 4 (cold tumor). (E) sXbp1/uXbp1 ratio, Ddit3, Hspa5, Atf4 and Atf6 mRNA levels in DsRed+ tumor cells (upper panel) and tumor-infiltrating CD8+ T cells (lower panel) in C57BL/6 mice bearing DsRed-MC38. Data are expressed as means ± SD (n=3). *P<0.05, **P<0.01.

We then confirmed the *in vivo* effect of SCD1 inhibition on CCL4 production in tumor cells using mice implanted with DsRed-labeled MC38 tumors. An increase of CCL4 along with a decrease of ATF3 was observed in DsRed^+^ MC38 cells isolated from tumors of mice treated with the SCD1 inhibitor (figure 4A, upper panel). These results suggest that SCD1 inhibition acts directly on tumor cells to enhance the production of CCL4 via the β-catenin/ATF3 axis, which results in the enhanced recruitment of DCs into tumors and the subsequent CD8^+^ T cell induction and accumulation.

We also evaluated the possible roles of SCD1 in the tumor immune microenvironment in human colon cancer. Transcriptome analysis of 57 advanced colon cancer samples revealed that one of the non-T cell inflamed subtypes (group 4) had low expression of *CD8a*, *IFNG* and *GZMB* with high expression of *SCD1* and *CTNNB1*(β-catenin) and its downstream molecules including *VEGFA* and *TCF12*. In contrast, the T cell inflamed subtype (group 1) showed high expression of *CD8a* and *CCL4* and low expression of *CTNNB1* and *SCD1* (figure 4B, C). This subtype also expressed molecules related to DCs with cross-priming ability, including *CD141* and *XCR1*, and the DC recruiting chemokine *CCL4* (figure 4C). *SCD1* expression was significantly correlated with β-catenin expression that was inversely correlated with *CD8a* and *XCR1* (figure 4D) as previously reported in human melanoma.^18^ These results indicate that the SCD1/β-catenin/CCL4 pathway may also be involved in the non-T cell inflamed subtype of human colon cancer.

### The SCD1 inhibition enhances CCL4 expression in T cells via reducing ER stress

In DsRed-labeled MC38-bearing mice, the expression of CCL4 was found to be significantly higher in tumor-infiltrating CD8^+^ T cells on a per cell basis than in tumor cells, and it was increased by treatment with the SCD1 inhibitor (figure 4A, lower panel). In CD8^+^ T cells, the expression of β-catenin and its downstream genes were relatively low at basal levels, and treatment with SCD1 inhibitors did not alter the β-catenin-related genes such as Tcf7 and Vegfa (online supplemental figure 5). ATF3, which regulates CCL4 expression, was reported to be induced by ER stress^26^ that is regulated by SCD1.^10^ Thus, we evaluated the status of ER stress in the tumor-infiltrating CD8^+^ T cells and found that administration of the SCD1 inhibitor reduced the expression of ATF3 (figure 4A, lower panel) accompanied by a reduction of ER stress-related molecules such as ratio of spliced Xbp1 (sXbp1; active isoform)/unspliced Xbp1 (uXbp1), Ddit3, Hspa5 (BiP), Atf4 and Atf6 (figure 4E lower panel), indicating that SCD1 inhibition reduced various ER stress pathways in CD8^+^ T cells. In contrast, SCD1 inhibition enhanced ER stress in MC38 tumor cells as previously reported^10^ (figure 4E upper panel), indicating that SCD1 had opposite *in vivo* effects on ER stress in tumor cells and in CD8^+^ T cells in mice treated with the SCD1 inhibitor. Therefore, different mechanisms through β-catenin or ER stress are employed for the SCD1 dependent regulation of CCL4 in cancer cells and in CD8^+^ T cells. These results indicate that in addition to enhancing CCL4 production in tumor cells, the SCD1 inhibitor also induce the high production of CCL4 by tumor infiltrating CD8^+^ T cells through the reduction of ER stress, suggesting that triggering of DC recruitment and subsequent T cell induction by CCL4 derived from tumor cells, and further amplification of antitumor T cell responses by CCL4 derived from tumor infiltrating CD8^+^ T cells.

### The SCD1 inhibitor acts directly on CD8^+^ T cells and DCs to enhance their functions

**Figure 5:**
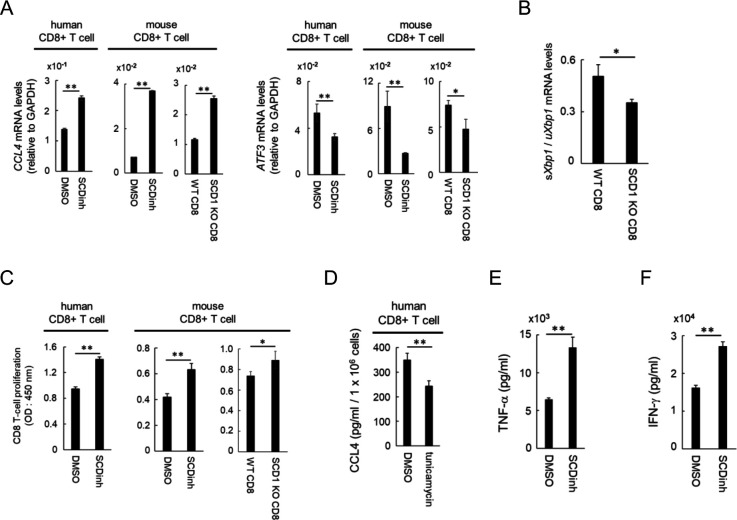
A SCD1 inhibitor directly enhances the function of CD8 + T cells and DCs *in vitro*. (A-C) CD8+ T cells were isolated from human PBMCs and spleens of wild-type (WT) or SCD1 knockout (KO) mice using MACS and were activated with an anti-CD3 monoclonal antibody, with an anti-CD28 monoclonal antibody and with IL-2. CD8+ T cells were cultured in RPMI medium containing 2% serum, after which DMSO or 1 µM SCD inhibitor (SCDinh) was added 2 days later, and cells were collected on day 4. (A) CCL4 and ATF3 expression levels in human and mouse CD8+ T cells. (B) sXbp1 / uXbp1 ratio in SCD1 KO mice CD8+ T cells. (C) Effect of SCD1 depletion on the proliferation of human and mouse CD8+ T cells evaluated by WST-1 assay. (D) Human CD8+ T cells were treated with DMSO or tunicamycin as described in materials and methods. On day 3, supernatants were collected and CCL4 levels were measured by ELISA. (E, F) Human DCs were differenciated from CD14+ PBMCs as described in the Methods. Differentiated DCs were activated by LPS stimulation. (E) TNF-α levels in the supernatant the day after LPS stimulation were evaluated by ELISA. (F) Activated DCs and allogenic CD8+ T cells were co-cultured and IFN-γ levels in the supernatant were measured the following day by ELISA. Data are expressed as means ± SD (n=3). *P<0.05, **P<0.01. SCDinh, SCD1 inhibitor; DMSO, dimethylsulfoxide control.

We then tested whether the inhibition of SCD1 acts directly on CD8^+^ T cells *in vitro*. CCL4 expression was increased along with a decrease of ATF3 in human peripheral blood and in mouse splenic CD8^+^ T cells treated *in vitro* with the SCD1 inhibitor (figure 5A). Similarly, higher CCL4 and lower ATF3 expression in CD8^+^ T cells obtained from spleens of SCD1 knockout (KO) mice were observed than those in CD8^+^ T cells from wild-type (WT) mice (figure 5A). Furthermore, as shown in the DsRed-MC38 model (figure 4E, lower panel), a ratio of sXbp1/uXbp1 was significantly lower in CD8^+^ T cells of SCD1 KO mice, indicating lower levels of ER stress in SCD1 negative CD8^+^ T cells (figure 5B). Proliferation of these human and mouse CD8^+^ T cells was significantly enhanced by SCD1 inhibition or by SCD1 KO (figure 5C). To further clarify the causal relationship between ER stress and CCL4 production, we tested the effect of ER stress inducer tunicamycin on human CD8^+^ T cells *in vitro* and found that it significantly reduced CCL4 production (figure 5D), indicating CCL4 production may also be regulated by ER stress in CD8^+^ T cells. Since SCD1 is also expressed in human DCs (online supplemental figure 6), we also analyzed the direct effect of the SCD1 inhibitor on human DCs. *In vitro* treatment of human monocyte derived DCs with the SCD1 inhibitor significantly increased the production of tumor necrosis factor (TNF)-α in the presence of lipopolysaccharide (figure 5E) and enhanced allogeneic T cells to produce IFN-γ (figure 5F). These results indicate that the SCD1 inhibitor acts directly on human CD8^+^ T cells and DCs and enhances their functions.

### SCD1 is a potential target to enhance the antitumor effects of an anti-PD-1 antibody

**Figure 6:**
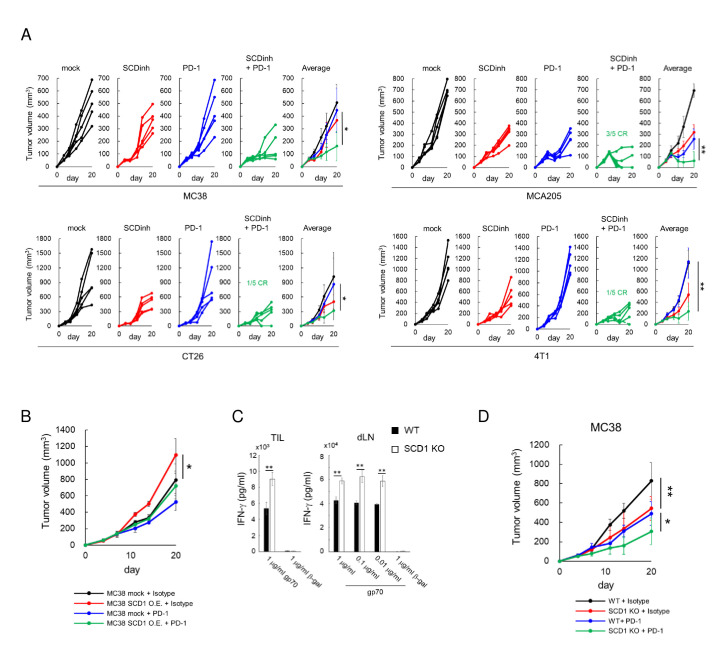
Inhibition of SCD1 enhances the therapeutic effect of anti-PD-1 antibodies. (A) Mice bearing MC38, MCA205, CT26 and 4T1 tumors were treated with a SCD1 inhibitor (SCDinh; 10 mg/kg) or with vehicle and with an anti-PD-1 antibody (200 µg /mouse) or an isotype-matched antibody. Tumor-growth curves for individual mice (left 4 panels) and average tumor volumes (right panel) (means ± SD; n=5). (B) C57BL/6 mice bearing vector control MC38 (MC38 mock) or SCD1 over-expressed MC38 tumors were treated with an anti-PD-1 (200 µg /mouse) or an isotype-matched antibody. (C) Antigen (gp70)‐specific T‐cell induction in SCD1 KO mice bearing MC38 tumors. (D) Wild-type (WT) or SCD1 KO mice bearing MC38 tumors were treated with an anti-PD-1 (200 µg /mouse) or an isotype-matched antibody.

Based on the increase of PD-1 positive CD8^+^ T cells in tumors in SCD1 inhibitor-treated mice (figure 2B, online supplemental figure 4A, right panel), we evaluated the antitumor effect of a combination of the SCD1 inhibitor and an anti-PD-1 antibody, and synergistic antitumor effects were observed in four tumor models. Some of the mice had complete tumor regression in MCA205, CT26 and 4T1 tumor models (figure 6A). Over-expression of SCD1 by lentiviral cDNA transduction resulted in significant increase of tumor growth compared to mock transfected MC38 (figure 6B), although anti-PD-1 antibody was still effective. Conversely, SCD1 knockdown MC38 by shRNA showed reduced tumor growth, although not significant possibly due to the redundant expression of SCD isoforms SCD2-4 (online supplemental figure 7). With the results of the increased CCL4 production by SCD1 knockdown tumor cells (figure 3B), these results suggested the immunoresistant role of SCD1 in tumor cells. In addition, the antitumor effect of the anti-PD-1 antibody was significantly higher in SCD1 KO mice accompanied by enhanced gp70-specific CD8^+^ T cell induction compared with WT mice (figure 6C, D). With the results of *in vitro* direct enhancing effects of the SCD1 inhibitor on T cells and DCs (figure 5C–F), these results indicate the immunosuppressive roles of SCD1 in host immune cells. Altogether, SCD1 inhibitors may be useful for combination immunotherapy with anti-PD-1/PD-L1 antibodies by acting on both cancer cells and immune cells.

### SCD1 and related free fatty acids are potential biomarkers to predict PD-1 antibody responses

**Figure 7:**
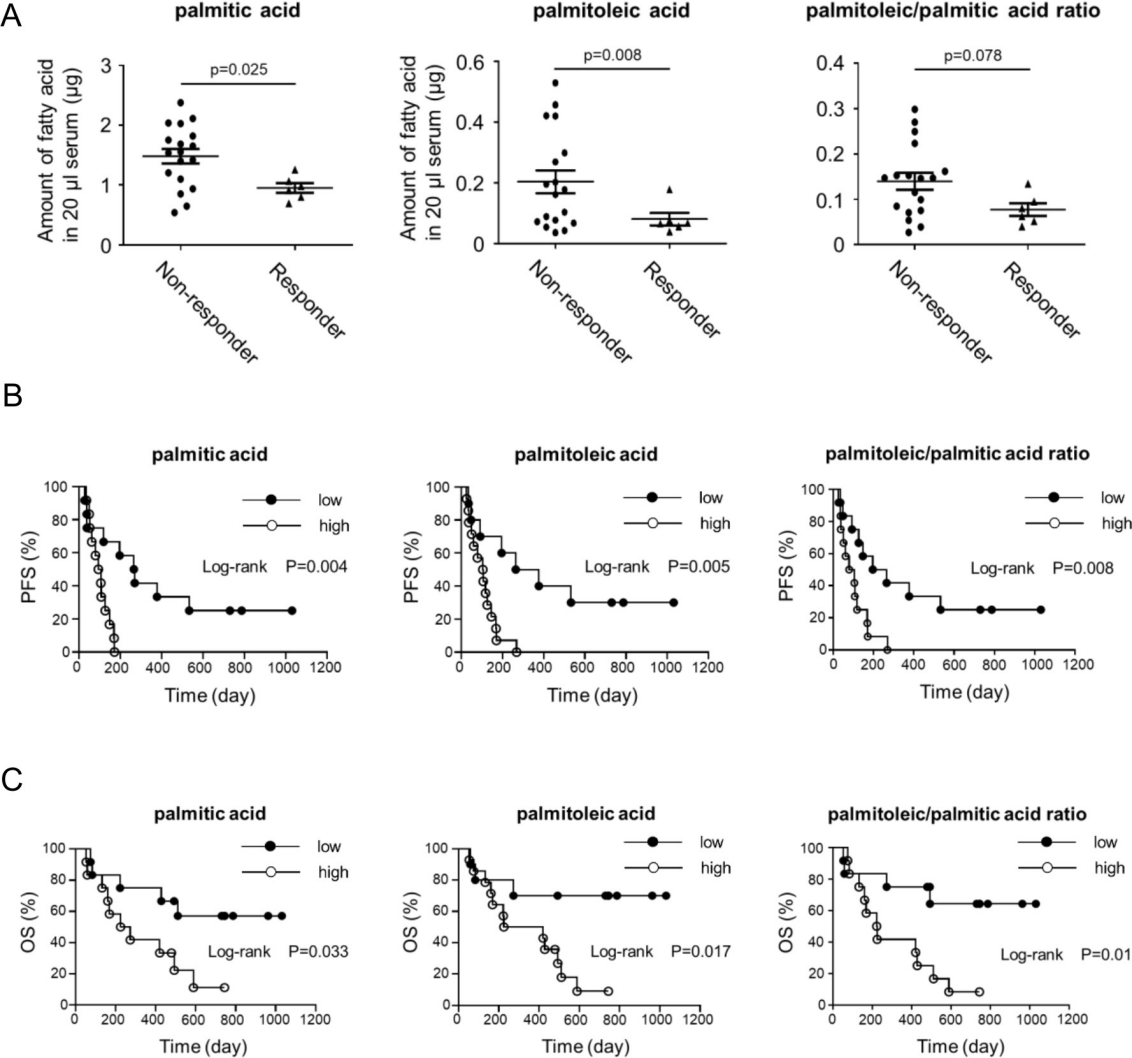
SCD1-related free fatty acids and ratio are potential biomarkers to predict PD-1 antibody responses and prognosis. (A) Pretreatment serum levels of palmitic and palmitoleic acid and ratio of palmitoleic/palmitic acid from NSCLC patients who subsequently did (Responder, n=6) or did not (Non-responder, n=18) respond to anti-PD-1 antibody treatment. (B, C) Kaplan–Meier analyses of NSCLC patients before anti-PD-1 antibody treatment. Kaplan–Meier curve of high (n=12) and low (n=12) palmitic acid, palmitoleic acid and palmitoleic/palmitic acid ratio in the serum of PFS patients (B) (HR 0.34, 95% CI 0.13 to 0.85; HR 0.32, 95% CI 0.13 to 0.77; HR 0.35, 95% CI 0.14 to 0.87, respectively) or OS patients (C) (HR 0.34, 95% CI 0.12 to 0.94; HR 0.25, 95% CI 0.09 to 0.69; HR 0.25, 95% CI 0.09 to 0.71, respectively). The threshold between high and low was median (palmitic acid; 1.33, palmitoleic acid; 0.10, palmitoleic/palmitic acid ratio; 0.10).

We also investigated the possible roles of SCD1 and related metabolites as biomarkers for immunotherapy. In addition to the relatively high expression level of SCD1 in non-T cell inflamed tumors that may not be sensitive to anti-PD-1 antibody therapy (figure 4B–D), fatty acid metabolites generated by SCD1 might be biomarkers for T cell dependent immunotherapy. We then evaluated SCD1-related free fatty acids at baseline (before the therapy) in the sera of non-small cell lung cancer (NSCLC) patients treated with anti-PD-1 antibody therapy and found that lower ratio of palmitoleic/palmitic acid and lower levels of serum palmitoleic acid and palmitic acid at baseline were significantly correlated with better responses and prognosis including progression-free survival (PFS) and overall survival (OS) following anti-PD-1 antibody treatment, although the ratio of oleic acid/stearic acid was not significanty correlated with the clinical outcomes (figure 7, online supplemental figure 8A–C). These results indicate that serum palmitoleic acid and palmitic acid regulated by SCD1 could be predictive biomarkers for immunotherapy.

## Discussion

The antitumor effects of PD-1/PD-L1 inhibitors alone are limited, and there is thus an urgent need to identify biomarkers to predict responses and therapeutic targets to develop more effective combination immunotherapies. In this study, we demonstrated that SCD1 in tumor cells suppresses antitumor CD8^+^ T cells through decreased numbers of DCs in tumors via the reduced level of DC-recruiting CCL4 caused by activation of the β-catenin pathway. In addition, we also showed that SCD1 was involved in the direct suppression of functions of CD8^+^ T cells and DCs, particularly the suppression of CCL4 production by CD8^+^ T cells through augmenting ER stress. Finally, we showed that SCD1 was an attractive target for combination immunotherapy because treatment with a SCD1 inhibitor augmented the antitumor effects of anti-PD-1 antibody, and SCD1 was a potential biomarker as suggested by high expression of SCD1 in non-T cell inflamed human colon cancers and the correlation of serum SCD1-related fatty acids with the responses to anti-PD-1 antibody in NSCLC patients.

Chemokines such as CCL4 have been reported to be important factors for developing a T cell-inflamed TME by recruiting DCs into tumors, which subsequently induce tumor antigen-specific CD8^+^ T cells and support tumor-infiltrating CD8^+^ effector T cells.^6^ In this study, we revealed two different mechanisms by which SCD1 inhibition enhances the production of CCL4 in cancer cells or CD8^+^ T cells. In cancer cells, SCD1 was found to inhibit the production of CCL4 that is negatively regulated by activated ATF3 through β-catenin signaling that is partly regulated by unsaturated fatty acids such as oleic acid and palmitoleic acid produced by SCD1.^18 24 25 27 28^ We have shown that SCD1 inhibition suppresses the expression of β-catenin and its downstream molecule, ATF3, and enhances the expression of CCL4 in mouse and human cancer cells and that SCD1 and the β-catenin pathway are positively correlated in patients with colorectal cancer.

In line with our findings, previous reports showed the positive relationship between SCD1 and Wnt/β-catenin signaling in human hepatocellular carcinoma and clear cell renal cell carcinoma cells. MUFA (palmitoleic acid) synthesized by SCD1 is ligated to Wnt protein, leading to its activation of β-catenin signaling.^27–29^ Oleic acid produced by SCD1 and its downstream metabolites, polyunsaturated 18-carbon fatty acids, stabilize β-catenin in cancer cells by preventing its proteasomal degradation.^25^ These observations suggest that SCD1 inhibition suppresses Wnt/β-catenin signaling in cancer cells through multiple mechanisms. Consistent with previous reports,^17^ we have also shown that SCD1 works as a downstream of β-catenin signaling. Therefore, SCD1 and β-catenin bidirectionally regulate the immunoresistance mechanism. The inhibition of SCD1 depletes oleic acid, which is the main component of the cell membrane, leading to cancer cell death^10^ that may also enhance the antitumor immune response via releasing tumor antigens to DCs. There are currently no clinically approved Wnt/β-catenin inhibitors due to their relatively poor specific activities and adverse effects. SCD1 inhibitors may be useful to restore antitumor immune responses for cancers with activated β-catenin signaling.

In contrast to cancer cells, SCD1 inhibited the production of CCL4 by CD8^+^ T cells through increased ATF3 activated by ER stress. We showed that SCD1 inhibition in CD8^+^ T cells reduced ER stress as shown by the decreased ratio of sXbp1/uXbp1 and other related molecules and increased CCL4 expression through ATF3 decrease *in vitro* and *in vivo*, without affecting β-catenin signaling. ATF3 has been previously reported to be regulated by ER stress^30^ and by β-catenin signaling.^18^ We showed that the SCD1 inhibitor might act directly on CD8^+^ T cells and DCs to enhance their functions with the reduced ER stress. It was reported that DC-intrinsic XBP1 promotes ovarian cancer progression and that silencing XBP1 in DCs enhances T cell antitumor immunity.^31^ XBP1 also enhances the expression of T cell exhaustion markers such as PD-1, TIM-3 and Lag3 in CD8^+^ T cells during infection,^20 32^ and the activation of XBP1 in T cells was associated with the suppression of mitochondrial activity and reduced IFN-γ, TNF-α and granzyme B production.^20 33^ Therefore, functions of immune cells, including CCL4 production, are regulated by ER stress, which can be a therapeutic target.

Additional lipid metabolism may be involved in the mechanisms by which SCD1 might suppress the functions of T cells or DCs. We observed decreased expression levels of acetyl-CoA carboxylase (ACC) and fatty acid synthase (FASN) in SCD1 inhibitor-treated mouse CD8^+^ T cells (online supplemental figure 9A, B) as previously reported.^34^ It was reported that fatty acid synthases via ACC and FASN are involved in abnormal fatty acid accumulation and suppress mitochondrial function in mouse DCs.^14^ Large amounts of saturated fatty acids have been reported to enhance ER stress in normal human and mouse cells such as muscle cells and renal epithelial cells through activation of the ER stress sensors IRE1α and PERK.^35 36^ Although SCD1 inhibition suppress conversion of saturated fatty acids to unsaturated fatty acids, decrease of total saturated fatty acids in CD8^+^ T cells caused by decrease of total fatty acids through decreased ACC and FASN, which were observed in CD8^+^ T cells treated with the SCD1 inhibitor, might result in the decreased ER stress in CD8^+^ T cells following SCD1 inhibitor treatment and enhanced T cell responses. These lipid metabolic enzymes are suppressed by SCD1 inhibition,^37^ possibly leading to enhanced antitumor immune responses. It has recently been reported that imbalance between unsaturated and saturated fatty acids by SCD1 activation and its downstream lipid metabolisms is involved in the tumor growth during calorie restriction.^38^ SCD1 inhibition may also activate CD8^+^ T cells through the change of cholesterol metabolism. Increase of intracellular cholesterol via inhibition of acetyl-coenzyme A acetyltransferase 1 (ACAT1) that uses oleic acid produced by SCD1 as a substrate for the esterification of cholesterol. ACAT1 inhibition was reported to activate the antitumor activity of CD8^+^ T cells.^39^

The mechanisms of opposite effects on ER stress in tumor cells and CD8^+^ T cells clearly observed *in vivo* following administration of the SCD1 inhibitor were not completely understood. It has been reported that SCD1 inhibition increases ER stress partly due to accumulation of SCD1 substrates such as palmitic acids and stearic acids, leading cell death of cancer cells.^10 15 40^ Different fatty acid metabolisms among cell types have been reported, including different effects of saturated fatty acids, utility of MUFAs, and expression of alternative fatty acid desaturase FADS2.^12 41 42^ In this study, decreased sXbp1/uXbp1 ratio in CD8^+^ T cells isolated from SCD1 KO mice and decrease of CCL4 production by *in vitro* treatment of CD8^+^ T cells with ER stress inducer tunicamycin suggest that SCD1 inhibition promoted CCL4 production partly via decreased ER stress in CD8^+^ T cells. In addition, improved conditions in immune-environment of tumors and lymph nodes may also be one of the mechanisms for the decrease of ER stress in CD8^+^ T cells *in vivo* following the SCD1 inhibitor administration.

In this study, the SCD1 inhibitor enhanced CCL4 production by cancer cells and by tumor-infiltrating CD8^+^ T cells. Higher production of CCL4 by tumor-infiltrating T cells was observed compared with cancer cells on a per-cell basis. CD8^+^ T cells themselves were reported to promote the recruitment of activated CD8^+^ T cells into tumors by recruiting various DCs via CCL3, CCL4 and XCL1.^43^ CCL4 from tumor cells may be essential in triggering the early phase of DC recruitment, while CCL4 from tumor-infiltrating CD8^+^ cells may further amplify the accumulation of DCs and T cells later as a positive feedback system for antitumor T cell responses.

Biomarkers for predicting responses to immune checkpoint inhibitors such as cancer gene alterations and immune responses have been extensively investigated using patients’ tumor and blood samples.^44–47^ In this study, we demonstrated that palmitic and palmitoleic acid levels and ratio of palmitoleic/palmitic acid in the sera of NSCLC patients at baseline were inversely correlated with the responses and prognosis including PFS and OS of patients treated with anti-PD-1 antibody and could be predictive biomarkers for PD-1/PD-L1 based immunotherapy. It is consistent with the negative roles of SCD1 shown in our mouse tumor models. It may be a reflection of SCD1 expression in cancer cells and immune cells in tumors. SCD1 was highly expressed in non-T cell inflamed subtype in colon cancer, which are likely to be resistant to anti-PD-1 antibody. Previous studies of patients with diabetes showed a correlation between the serum SCD1-related fatty acid ratio^48^ and SCD1 gene expression in tissues. Although further study is needed to confirm those biomarker roles in patients with various types of cancers, SCD1-related free fatty acids may be potential biomarkers for cancer immune therapies. Interestingly, no significant correlation between the ratio of serum oleic acid and stearic acid and clinical outcomes was observed in this study possibly due to the involvement of additional systemic fatty acid metabolisms of serum oleic acid and stearic acid in patients, regardless of possible changes of their free fatty acids in tumor microenvironments as shown in mouse models. In addition, low levels of palmitic acid and stearic acid, substrates of SCD1 appeared to correlate with clinical outcomes, indicating fatty acid synthesis via ACC and FASN in the downstream of SCD1 may also be involved in the immunosuppressive conditions in patients with cancer as shown in our mouse models in this study. Further mechanistic investigation is needed for their precise mechanisms. Altogether, we have shown in this study that SCD1 expressed in cancer cells and immune cells such as CD8^+^ T cells and DCs is involved in the immune-resistant mechanisms for immune checkpoint blockade and is an attractive target for the development of new diagnostic and therapeutic strategies for effective cancer immunotherapy.

10.1136/jitc-2022-004616.supp1 Supplementary data

10.1136/jitc-2022-004616.supp2 Supplementary data

10.1136/jitc-2022-004616.supp3 Supplementary data

10.1136/jitc-2022-004616.supp4 Supplementary data

10.1136/jitc-2022-004616.supp5 Supplementary data

## Data Availability

Data are available on reasonable request. All data relevant to the study are included in the article or uploaded as supplementary information.

## References

[1] Robert C, Long GV, Brady B, et al. Nivolumab in previously untreated melanoma without BRAF mutation. N Engl J Med 2015;372:320–30. 10.1056/NEJMoa1412082 25399552

[2] Hodi FS, O'Day SJ, McDermott DF, et al. Improved survival with ipilimumab in patients with metastatic melanoma. N Engl J Med 2010;363:711–23. 10.1056/NEJMoa1003466 20525992 PMC3549297

[3] Le DT, Durham JN, Smith KN, et al. Mismatch repair deficiency predicts response of solid tumors to PD-1 blockade. Science 2017;357:409–13. 10.1126/science.aan6733 28596308 PMC5576142

[4] Spranger S, Gajewski TF. Impact of oncogenic pathways on evasion of antitumour immune responses. Nat Rev Cancer 2018;18:139–47. 10.1038/nrc.2017.117 29326431 PMC6685071

[5] Ayers M, Lunceford J, Nebozhyn M, et al. IFN-γ-related mRNA profile predicts clinical response to PD-1 blockade. J Clin Invest 2017;127:2930–40. 10.1172/JCI91190 28650338 PMC5531419

[6] Harlin H, Meng Y, Peterson AC, et al. Chemokine expression in melanoma metastases associated with CD8+ T-cell recruitment. Cancer Res 2009;69:3077–85. 10.1158/0008-5472.CAN-08-2281 19293190 PMC3886718

[7] Melero I, Berman DM, Aznar MA, et al. Evolving synergistic combinations of targeted immunotherapies to combat cancer. Nat Rev Cancer 2015;15:457–72. 10.1038/nrc3973 26205340

[8] Bai Y, McCoy JG, Levin EJ, et al. X-ray structure of a mammalian stearoyl-CoA desaturase. Nature 2015;524:252–6. 10.1038/nature14549 26098370 PMC4689147

[9] Hodson L, Fielding BA. Stearoyl-CoA desaturase: rogue or innocent bystander? Prog Lipid Res 2013;52:15–42. 10.1016/j.plipres.2012.08.002 23000367

[10] Igal RA. Stearoyl CoA desaturase-1: new insights into a central regulator of cancer metabolism. Biochim Biophys Acta 2016;1861:1865–80. 10.1016/j.bbalip.2016.09.009 27639967

[11] Peck B, Schug ZT, Zhang Q, et al. Inhibition of fatty acid desaturation is detrimental to cancer cell survival in metabolically compromised environments. Cancer Metab 2016;4:6. 10.1186/s40170-016-0146-8 27042297 PMC4818530

[12] Minville-Walz M, Pierre A-S, Pichon L, et al. Inhibition of stearoyl-CoA desaturase 1 expression induces CHOP-dependent cell death in human cancer cells. PLoS One 2010;5:e14363. 10.1371/journal.pone.0014363 21179554 PMC3002938

[13] Ariyama H, Kono N, Matsuda S, et al. Decrease in membrane phospholipid unsaturation induces unfolded protein response. J Biol Chem 2010;285:22027–35. 10.1074/jbc.M110.126870 20489212 PMC2903364

[14] Mogilenko DA, Haas JT, L'homme L, et al. Metabolic and innate immune cues merge into a specific inflammatory response via the UPR. Cell 2019;177:1201–16. 10.1016/j.cell.2019.03.018 31031005

[15] Leung JY, Kim WY. Stearoyl Co-a desaturase 1 as a ccRCC therapeutic target: death by stress. Clin Cancer Res 2013;19:3111–3. 10.1158/1078-0432.CCR-13-0800 23709675 PMC3898854

[16] Mauvoisin D, Charfi C, Lounis AM, et al. Decreasing stearoyl-CoA desaturase-1 expression inhibits β-catenin signaling in breast cancer cells. Cancer Sci 2013;104:36–42. 10.1111/cas.12032 23013158 PMC7657162

[17] Lai KKY, Kweon S-M, Chi F, et al. Stearoyl-Coa desaturase promotes liver fibrosis and tumor development in mice via a Wnt positive-signaling loop by stabilization of low-density Lipoprotein-Receptor-Related proteins 5 and 6. Gastroenterology 2017;152:1477–91. 10.1053/j.gastro.2017.01.021 28143772 PMC5406249

[18] Spranger S, Bao R, Gajewski TF. Melanoma-intrinsic β-catenin signalling prevents anti-tumour immunity. Nature 2015;523:231–5. 10.1038/nature14404 25970248

[19] Yaguchi T, Goto Y, Kido K, et al. Immune suppression and resistance mediated by constitutive activation of Wnt/β-catenin signaling in human melanoma cells. J Immunol 2012;189:2110–7. 10.4049/jimmunol.1102282 22815287

[20] Ma X, Bi E, Lu Y, et al. Cholesterol Induces CD8^+^ T Cell Exhaustion in the Tumor Microenvironment. Cell Metab 2019;30:143–56. 10.1016/j.cmet.2019.04.002 31031094 PMC7061417

[21] Cao Y, Trillo-Tinoco J, Sierra RA, et al. ER stress-induced mediator C/EBP homologous protein thwarts effector T cell activity in tumors through T-bet repression. Nat Commun 2019;10:1280. 10.1038/s41467-019-09263-1 30894532 PMC6426975

[22] Hurst KE, Lawrence KA, Essman MT, et al. Endoplasmic Reticulum Stress Contributes to Mitochondrial Exhaustion of CD8^+^ T Cells. Cancer Immunol Res 2019;7:476–86. 10.1158/2326-6066.CIR-18-0182 30659052 PMC6397687

[23] Xin Z, Zhao H, Serby MD, et al. Discovery of piperidine-aryl urea-based stearoyl-CoA desaturase 1 inhibitors. Bioorg Med Chem Lett 2008;18:4298–302. 10.1016/j.bmcl.2008.06.088 18632269

[24] Khuu CH, Barrozo RM, Hai T, et al. Activating transcription factor 3 (ATF3) represses the expression of CCl4 in murine macrophages. Mol Immunol 2007;44:1598–605. 10.1016/j.molimm.2006.08.006 16982098

[25] Kim H, Rodriguez-Navas C, Kollipara RK, et al. Unsaturated fatty acids stimulate tumor growth through stabilization of β-catenin. Cell Rep 2015;13:495–503. 10.1016/j.celrep.2015.09.010 26456834 PMC4618234

[26] Mungrue IN, Pagnon J, Kohannim O, et al. CHAC1/MGC4504 is a novel proapoptotic component of the unfolded protein response, downstream of the ATF4-ATF3-CHOP cascade. J Immunol 2009;182:466–76. 10.4049/jimmunol.182.1.466 19109178 PMC2846782

[27] Takada R, Satomi Y, Kurata T, et al. Monounsaturated fatty acid modification of Wnt protein: its role in Wnt secretion. Dev Cell 2006;11:791–801. 10.1016/j.devcel.2006.10.003 17141155

[28] Rios-Esteves J, Resh MD. Stearoyl CoA desaturase is required to produce active, lipid-modified Wnt proteins. Cell Rep 2013;4:1072–81. 10.1016/j.celrep.2013.08.027 24055053 PMC3845236

[29] Stoffel W, Schmidt-Soltau I, Jenke B, et al. Hair Growth Cycle Is Arrested in SCD1 Deficiency by Impaired Wnt3a-Palmitoleoylation and Retrieved by the Artificial Lipid Barrier. J Invest Dermatol 2017;137:1424–33. 10.1016/j.jid.2017.02.973 28259688

[30] Jiang H-Y, Wek SA, McGrath BC, et al. Activating transcription factor 3 is integral to the eukaryotic initiation factor 2 kinase stress response. Mol Cell Biol 2004;24:1365–77. 10.1128/MCB.24.3.1365-1377.2004 14729979 PMC321431

[31] Cubillos-Ruiz JR, Silberman PC, Rutkowski MR, et al. Er stress sensor XBP1 controls anti-tumor immunity by disrupting dendritic cell homeostasis. Cell 2015;161:1527–38. 10.1016/j.cell.2015.05.025 26073941 PMC4580135

[32] Kamimura D, Bevan MJ. Endoplasmic reticulum stress regulator XBP-1 contributes to effector CD8+ T cell differentiation during acute infection. J Immunol 2008;181:5433–41. 10.4049/jimmunol.181.8.5433 18832700 PMC2776092

[33] Song M, Sandoval TA, Chae C-S, et al. IRE1α-XBP1 controls T cell function in ovarian cancer by regulating mitochondrial activity. Nature 2018;562:423–8. 10.1038/s41586-018-0597-x 30305738 PMC6237282

[34] Jiang G, Li Z, Liu F, et al. Prevention of obesity in mice by antisense oligonucleotide inhibitors of stearoyl-CoA desaturase-1. J Clin Invest 2005;115:1030–8. 10.1172/JCI200523962 15761499 PMC1062893

[35] Salvadó L, Coll T, Gómez-Foix AM, et al. Oleate prevents saturated-fatty-acid-induced ER stress, inflammation and insulin resistance in skeletal muscle cells through an AMPK-dependent mechanism. Diabetologia 2013;56:1372–82. 10.1007/s00125-013-2867-3 23460021

[36] Volmer R, van der Ploeg K, Ron D. Membrane lipid saturation activates endoplasmic reticulum unfolded protein response transducers through their transmembrane domains. Proc Natl Acad Sci U S A 2013;110:4628–33. 10.1073/pnas.1217611110 23487760 PMC3606975

[37] Geng F, Cheng X, Wu X, et al. Inhibition of SOAT1 suppresses glioblastoma growth via blocking SREBP-1-mediated lipogenesis. Clin Cancer Res 2016;22:5337–48. 10.1158/1078-0432.CCR-15-2973 27281560 PMC5093025

[38] Lien EC, Westermark AM, Zhang Y, et al. Low glycaemic diets alter lipid metabolism to influence tumour growth. Nature 2021;599:302–7. 10.1038/s41586-021-04049-2 34671163 PMC8628459

[39] Yang W, Bai Y, Xiong Y, et al. Potentiating the antitumour response of CD8(+) T cells by modulating cholesterol metabolism. Nature 2016;531:651–5. 10.1038/nature17412 26982734 PMC4851431

[40] Roongta UV, Pabalan JG, Wang X, et al. Cancer cell dependence on unsaturated fatty acids implicates stearoyl-CoA desaturase as a target for cancer therapy. Mol Cancer Res 2011;9:1551–61. 10.1158/1541-7786.MCR-11-0126 21954435

[41] Vriens K, Christen S, Parik S, et al. Evidence for an alternative fatty acid desaturation pathway increasing cancer plasticity. Nature 2019;566:403–6. 10.1038/s41586-019-0904-1 30728499 PMC6390935

[42] Deldicque L, Van Proeyen K, Francaux M, et al. The unfolded protein response in human skeletal muscle is not involved in the onset of glucose tolerance impairment induced by a fat-rich diet. Eur J Appl Physiol 2011;111:1553–8. 10.1007/s00421-010-1783-1 21188411

[43] Brewitz A, Eickhoff S, Dähling S, et al. CD8^+^ T Cells Orchestrate pDC-XCR1^+^ Dendritic Cell Spatial and Functional Cooperativity to Optimize Priming. Immunity 2017;46:205–19. 10.1016/j.immuni.2017.01.003 28190711 PMC5362251

[44] Snyder A, Makarov V, Merghoub T, et al. Genetic basis for clinical response to CTLA-4 blockade in melanoma. N Engl J Med 2014;371:2189–99. 10.1056/NEJMoa1406498 25409260 PMC4315319

[45] Rizvi NA, Hellmann MD, Snyder A, et al. Cancer immunology. mutational landscape determines sensitivity to PD-1 blockade in non-small cell lung cancer. Science 2015;348:124–8. 10.1126/science.aaa1348 25765070 PMC4993154

[46] Le DT, Uram JN, Wang H, et al. PD-1 blockade in tumors with mismatch-repair deficiency. N Engl J Med 2015;372:2509–20. 10.1056/NEJMoa1500596 26028255 PMC4481136

[47] Hatae R, Chamoto K, Kim YH, et al. Combination of host immune metabolic biomarkers for the PD-1 blockade cancer immunotherapy. JCI Insight 2020;5. 10.1172/jci.insight.133501. [Epub ahead of print: 30 01 2020]. PMC7098729 31855576

[48] Risérus U, Tan GD, Fielding BA, et al. Rosiglitazone increases indexes of stearoyl-CoA desaturase activity in humans: link to insulin sensitization and the role of dominant-negative mutation in peroxisome proliferator-activated receptor-gamma. Diabetes 2005;54:1379–84. 10.2337/diabetes.54.5.1379 15855323

