# Supplementary material for: Inhibition of stearoyl-CoA desaturase 1 (SCD1) enhances the antitumor T cell response through regulating β-catenin signaling in cancer cells and ER stress in T cells and synergizes with anti-PD-1 antibody
Source: J Immunother Cancer. 2022 Jul 6;10(7):e004616. doi: 10.1136/jitc-2022-004616 (PMC9260842; doi:10.1136/jitc-2022-004616)
Supplement: Supplementary data [file jitc-2022-004616supp006.pdf]

BMJ Publishing Group Limited (BMJ) disclaims all liability and responsibility arising from any reliance placed on this supplemental material which has been supplied by the author(s)

Supplementary Files

Inhibition of stearoyl-CoA desaturase 1 (SCD1) enhances the anti-tumor T cell response through regulating β-catenin signaling in cancer cells and ER stress in T cells and synergizes with anti-PD-1 antibody

Supplementary materials and methods

Gp70-specific CD8 T cell induction

At day 20, whole cells from draining lymph nodes (1×10^7 cells) were restimulated with 1 μg/ml gp70 peptide, the H-2K^b-restricted T cell epitope peptide (MuLV gp70 p15E; aa604-611(KSPWFTTL)) for C57BL/6 mouse models (Medical & Biological Laboratories) for 5 days in RPMI 1640 with 10% FBS. Tumor-infiltrating CD8$^+$ T cells (1×10^6 cells) positively selected using CD8$^+$ Microbeads (Miltenyi Biotec) were co-cultured with syngeneic 32-Gy irradiated splenocytes (1×10^7 cells) in RPMI 1640 with 10% FBS and were re-stimulated with 1 μg/ml T cell epitope peptide for 2 days in a similar manner. T cells were collected after 2 or 5 days using Lympholyte-M (Cedarlane) and were co-cultured in 96-well plates in the presence of the T cell epitope peptide at concentrations of 0, 0.01, 0.1 and 1 mg/ml, with EL-4 (1×10^5 T cells and 2×10^5 EL-4/200 μl/well). IFN-γ concentrations were measured using an enzyme-linked immunosorbent assay (ELISA) after 24 h.

Katoh Y, *et al. J Immunother Cancer* 2022; 10:e004616. doi: 10.1136/jitc-2022-004616

Gene array analysis of tumor tissues from colon cancer patients

Fifty-seven patients with advanced or recurrent CRC who underwent surgical resection of a primary tumor between 1998 and 2010 were enrolled. All the patients provide written informed consent. Study approval was obtained from the ethics committees of Tohoku University, Sendai Open Hospital, South Miyagi Medical Center and National Cancer Center Hospital. Total RNAs were isolated from surgically resected FFPE tissues using a column-based method (Absolutely RNA FFPE Kit; Agilent Technologies). The purity and integrity of each RNA was evaluated using an ND-1000 Spectrophotometer (NanoDrop) and an Agilent 2100 Bioanalyzer (Agilent Technologies). Labeling and hybridization were performed using the Gene Expression Microarray Analysis of Archival FFPE Samples (Agilent Technologies). cDNAs were amplified from total RNA (300 ng) using a TransPlex Complete Whole Transcriptome Amplification Kit (Sigma). The amplified cDNAs were then purified using a QIAquick PCR Purification Kit (Qiagen). The purified cDNAs (1.5 μg) were labeled with Cy3 using a Genomic DNA ULS Labeling Kit (Agilent Technologies). Finally, 5 μl of 10× blocking agent and 25 μl of 2× GE hybridization buffer were added to dilute the labeled cDNA, and 40 μl of the hybridization solution were then dispensed into a gasket slide and assembled with the Agilent Whole Human Genome 4 × 44 K Microarray (Agilent Technologies). The slides were incubated for 17 h at 65°C in an Agilent hybridization oven and then washed at room temperature using the Gene

Expression Microarray Analysis of Archival FFPE Samples (Agilent Technologies). The hybridized arrays were immediately scanned using an Agilent Microarray Scanner D (Agilent Technologies). Intensity values of each scanned feature were quantified using Agilent Feature Extraction software (Agilent Technologies). The gene expression information has been registered in GEO (https://www.ncbi.nlm.nih.gov/geo/query/acc.cgi?acc=GSE104645). We applied rank-based quantile normalization to the raw signal data using GeneSpring software (Agilent Technologies). In this study, we focused on immune related genes, fatty acid metabolism related genes and β-catenin pathway genes that did not have high variance or low intensity of expression. We selected β-catenin pathway genes from the KEGG database (https://www.genome.jp/kegg/) and used the median expression value of those genes for subsequent analyses. The data were analyzed using unsupervised hierarchical clustering with Euclidean distance and average linkage method using the TIGR MultiExperimentViewer (MeV) tool (http://mev.tm4.org/).

RNA extraction and RT-qPCR analysis

Total RNA was isolated from tumors at day 20 post-inoculation *in vivo* and from cultured cells recovered *in vitro*, using an RNeasy Mini Kit (Qiagen). RT-qPCR was performed according to the standard $2^{-\Delta Ct}$ protocol. The relative quantification value is expressed as $2^{-\Delta Ct}$, in which ΔCt is the difference between the mean Ct value of triplicate measurements of the sample and the

endogenous GAPDH control. TaqMan RT-PCR primers and probes for human and mouse ATF3, CCL4, CD8 and human SCD1 were purchased from Applied Biosystems, and for mouse Acaca, Fasn, Ddit3, Hspa5, Atf4, Atf6, Tcf7, Vegfa, Ctnnb1 and Ccnd2 were purchased from Integrated DNA Technologies. SYBR Green RT-PCR primers for mouse Scd1, sXbp1 and uXbp1 were purchased from Sigma-Aldrich (Supplementary table. S1).[1]

Immunohistochemistry (IHC)

Tumor samples were obtained from mice 20 days after tumor inoculation. Formalin-fixed paraffin-embedded sections of mouse tissues were subjected to IHC analysis, as described previously.[2] The CD8α (D4W2Z) XP® rabbit mAb (mouse-specific) was purchased from Cell Signaling. Histofine simple stain MAX-PO (Nichirei Biosciences Inc.) was used to detect primary antibodies in mouse tissues, according to the manufacturer’s instructions. CD8-stained slides were scanned using a high-resolution digital slide scanner (NanoZoomer-XR C12000; Hamamatsu Photonics). The number of $CD8^+$ T cell infiltrates per 1 mm^2 of tumor was calculated automatically using a computerized image analysis system (Tissue Studio; Definiens).

Flow cytometry

Katoh Y, et al. J Immunother Cancer 2022; 10:e004616. doi: 10.1136/jitc-2022-004616

For gp70-specific $CD8^+$ T cell staining, cells were stained with anti-CD8 (fluorescein isothiocyanate (FITC); MBL), H-2Ld MuLV gp70 tetramer, H-2Ld β-galactosidase tetramer (phycoerythrin (PE); MBL), CD3 (Brilliant Violet 421; BioLegend) and CD45 (V500; BD Biosciences). For tumor-infiltrating $CD8^+$ T cell staining, cells were stained with anti-TIGIT (PE; eBioscience), CD4 (PE/Dazzle 594; BioLegend), Lag3 (PerCP/Cyanine5.5; BioLegend), 4-1BB (PE/Cy7; eBioscience), CD8 (Alexa700; BD Biosciences), CD3 (allophycocyanin (APC)/Cy7; BioLegend), PD-1 (Brilliant Violet 421; BioLegend), and CD45 (V500; BD Biosciences). For $CD45^+$ $CD11c^+$ cell staining, cells were stained with anti-I-A[d] (FITC; BD Biosciences), CD80 (PE; BioLegend), CD83 (APC; BD Biosciences), CD86 (APC/Cy7; BioLegend), CD11c (Brilliant Violet 421; BioLegend) and CD45 (V500; BD Biosciences). For Treg cell staining, cells were stained with anti-CD8 (FITC; MBL), Foxp3 (PE; BD Biosciences), CD4 (PE/Dazzle 594; BioLegend), CD25 (PE/Cy7; BD Biosciences), CD3 (Alexa700; BioLegend) and CD45 (V500; BD Biosciences). Intracellular staining of FOXP3 was performed using FOXP3 Fix/Perm Buffer (BioLegend) according to the manufacturer’s instructions. Flow cytometry sample acquisition was performed using a Gallios flow cytometer (Beckman Coulter) and analysis was performed using Kaluza software (Beckman Coulter).

Cell sorting

Katoh Y, *et al. J Immunother Cancer* 2022; 10:e004616. doi: 10.1136/jitc-2022-004616

In experiments using DsRed-MC38 cells, tumor samples were obtained from mice 20 days after tumor inoculation. The cells were stained with CD8 (Alexa700; BD Pharmingen), CD3 (APC/Cy7; BioLegend) and CD45 (Brilliant Violet 421; BioLegend). Cell sorting was performed using an FACSAria II (BD) and cells were collected in RPMI containing 10% FBS.

Gene knockdown or overexpression

Fifty to 100 nM target gene-specific or control small interfering RNAs (siRNAs) (Ambion or Invitrogen; shown in Supplementary table. S2) were introduced into cancer cells using Lipofectamine. Cells were collected for western blotting and qPCR after incubation for 72 h. The vector pLKO.1 including short hairpin RNA (shRNA) targeting mouse SCD1 gene (NM_009127) was obtained from Merck (TRCN0000114331). Empty pLKO.1 was used as negative control. The shRNA and two helper plasmids, pCMV-VSV-G-Rev (addgene) and pMDL-g/p-RRE (addgene), were transfected into 293T cells. HilyMax (Dojindo) was used to transfect the constructs. The supernatant was collected at 48 hours post-transfection and concentrated by polyethylene glycol precipitation using a Lenti-X™ Concentrator (Clontec Laboratories). The recombinant lentivirus-containing medium was added to MC38 cells. Overexpression of human β-catenin S37F mutation in 938mel cells and mouse SCD1 in MC38 cells were established by puromycin selection as previously described.[2]

Katoh Y, et al. J Immunother Cancer 2022; 10:e004616. doi: 10.1136/jitc-2022-004616

In vitro functional analysis of $CD8^+$ T cells

$CD8^+$ T cells were isolated from human peripheral blood mononuclear cells (PBMCs) and spleens from WT and SCD1 KO mice using magnetic-activated cell sorting (MACS) and were activated with an anti-CD3 monoclonal antibody (human: OKT3, mouse: 145-2C11, 2 mg/ml), anti-CD28 mAb (human: 9.3, mouse: PV-1, 2 mg/ml) and IL-2. $CD8^+$ T cells (1×10^5 cells) were cultured in RPMI medium containing 2% serum, DMSO or 1 μM SCD inhibitor (SCDinh) was added 2 days after stimulation, and cells were collected on day 4. $CD8^+$ T cell proliferation was evaluated using WST-1 Solution Reagent (Roche) according to the manufacturer's instructions.

Induction of ER stress in human $CD8^+$ T cells

$CD8^+$ T cells were isolated from human PBMCs using MACS and were activated with an anti-CD3 monoclonal antibody, with an anti-CD28 monoclonal antibody and with IL-2. $CD8^+$ T cells were cultured in AIM-V medium containing 10% human AB serum, after which DMSO or 1 μg/ml tunicamycin was added following day, and supernatant were collected on day 3.

In vitro functional analysis of DCs

8

Human PBMCs were obtained from healthy donors with informed consent. Human monocyte-derived DCs were generated as described previously.[3] Briefly, human CD14^{+} monocytes (1 × 10^{6} cells) were cultured with granulocyte-macrophage colony-stimulating factor and IL-4. On day 5, DCs were washed, reseeded and then stimulated with 1 μg/ml LPS with or without 1 μM SCD1 inhibitor. Culture supernatants were collected on day 6 to measure TNF-α activity by ELISA (BD Biosciences Pharmingen), and the DCs were further cultured for 3 days. DCs (0.3 × 10^{4} cells) and allogenic CD8^{+} T cells (1 × 10^{5} cells) were co-cultured on day 9 and supernatants were collected the next day to measure IFN-γ by ELISA (BD Biosciences Pharmingen).

Western blotting

Cytoplasmic samples were prepared using RIPA buffer (Thermo Scientific) containing cOmplete Mini EDTA-free Protease Inhibitor Cocktail (Roche) and nuclear samples were prepared using NE-PER Nuclear and Cytoplasmic Extraction Reagents (Thermo Scientific), according to the manufacturer’s instructions. Cell lysates were centrifuged at 14,000 × *g* for 30 min at 4°C and the protein concentration of each sample was determined using Bio-Rad Protein Assay Dye Reagent Concentrate (Bio-Rad), with bovine serum albumin (Bio-Rad) as a standard. Proteins were subjected to sodium dodecyl sulfate-polyacrylamide gel electrophoresis (Bio-Rad) and transferred to Immobilon-P membranes (Merck). Blots were blocked with

Blocking One™ (Nacalai) at room temperature for 60 min and then incubated with the following primary antibodies: mouse anti-SCD1 antibody (CD.E10; Abcam), rabbit anti-β-catenin antibody (D10A8; Cell Signaling), rabbit anti-GAPDH antibody (FL-335; Santa Cruz Biotechnology) and rabbit anti-lamin A/C antibody (Cell Signaling) overnight at 4°C. After washing three times with TBST (20 mM Tris–HCl, 150 mM NaCl and 0.02% Tween-20; pH 7.4), the blots were incubated with Goat Anti-Rabbit IgG H&L (Abcam) and Goat Anti-Mouse IgG H&L (Abcam) secondary antibodies at room temperature for 1 h. Signals were detected using SuperSignal West Femto Maximum Sensitivity Substrate (Thermo Scientific).

Statistical analysis

All statistical analyses were performed using GraphPad Prism 9 software. Comparisons between two groups were assessed using unpaired or paired (for matched comparisons) two-tailed Student's *t*-tests or non-parametric Mann–Whitney U-tests. Multiple comparisons were assessed by one-way ANOVA, including Tukey's multiple comparisons tests. Significance for pairwise correlation analysis was calculated using the Pearson correlation coefficient. Survival rates were compared using the log-rank test. Data are presented as means ± S.D. A $P<0.05$ is considered statistically significant.

References

1. Yoon SB, Park YH, Choi SA, et al. Real-time PCR quantification of spliced X-box binding protein 1 (XBP1) using a universal primer method. *PLoS One* 2019;14(7):e0219978. doi: 10.1371/journal.pone.0219978 [published Online First: 2019/07/23]

2. Kinoshita T, Sayem MA, Yaguchi T, et al. Inhibition of vascular adhesion protein-1 enhances the anti-tumor effects of immune checkpoint inhibitors. *Cancer Sci* 2021;112(4):1390-401. doi: 10.1111/cas.14812 [published Online First: 2021/01/17]

3. Yaguchi T, Goto Y, Kido K, et al. Immune suppression and resistance mediated by constitutive activation of Wnt/beta-catenin signaling in human melanoma cells. *Journal of immunology (Baltimore, Md : 1950)* 2012;189(5):2110-7. doi: 10.4049/jimmunol.1102282 [published Online First: 2012/07/21]

Supplementary figures

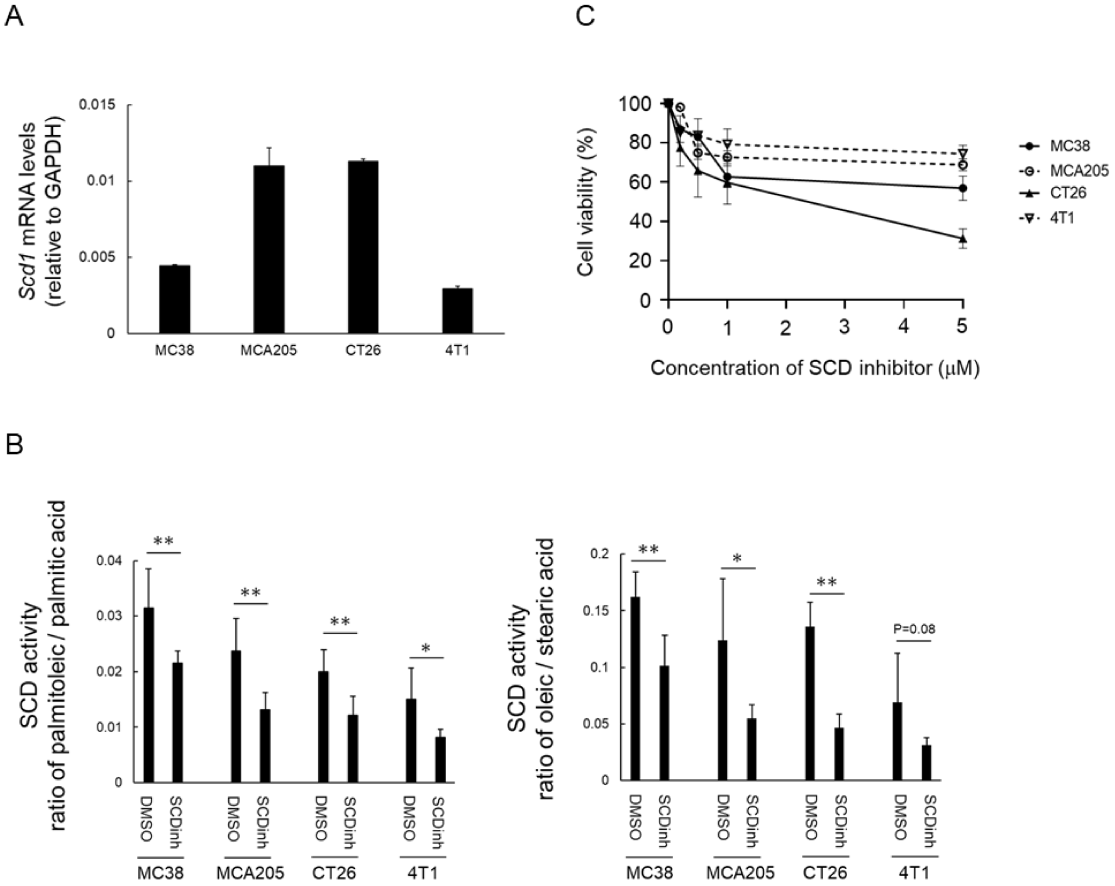

Supplementary Figure 1. SCD1 gene expression, enzyme activity and direct effect of SCD1 inhibitor in murine cancer cell lines. (A, B) Cancer cells were cultured in RPMI medium containing 2% FBS. **(A)** Total RNA was extracted and SCD1 mRNA levels were evaluated by real-time RT-PCR. **(B)** Cancer cells were treated with a SCD inhibitor (SCDinh) or DMSO. The metabolite to substrate ratios of SCD1, palmitoleic acid to palmitic acid and oleic acid to stearic acid in four cancer cells were determined. **(C)** Mice cancer cell lines were treated with

Katoh Y, *et al. J Immunother Cancer* 2022; 10:e004616. doi: 10.1136/jitc-2022-004616

SCD1inhibitor (0, 0.2, 0.5, 1, 5 μM). Cell viability was assessed by WST-1 assay. Data are expressed as means ± SD (n=5).

Katoh Y, *et al*. *J Immunother Cancer* 2022; 10:e004616. doi: 10.1136/jitc-2022-004616

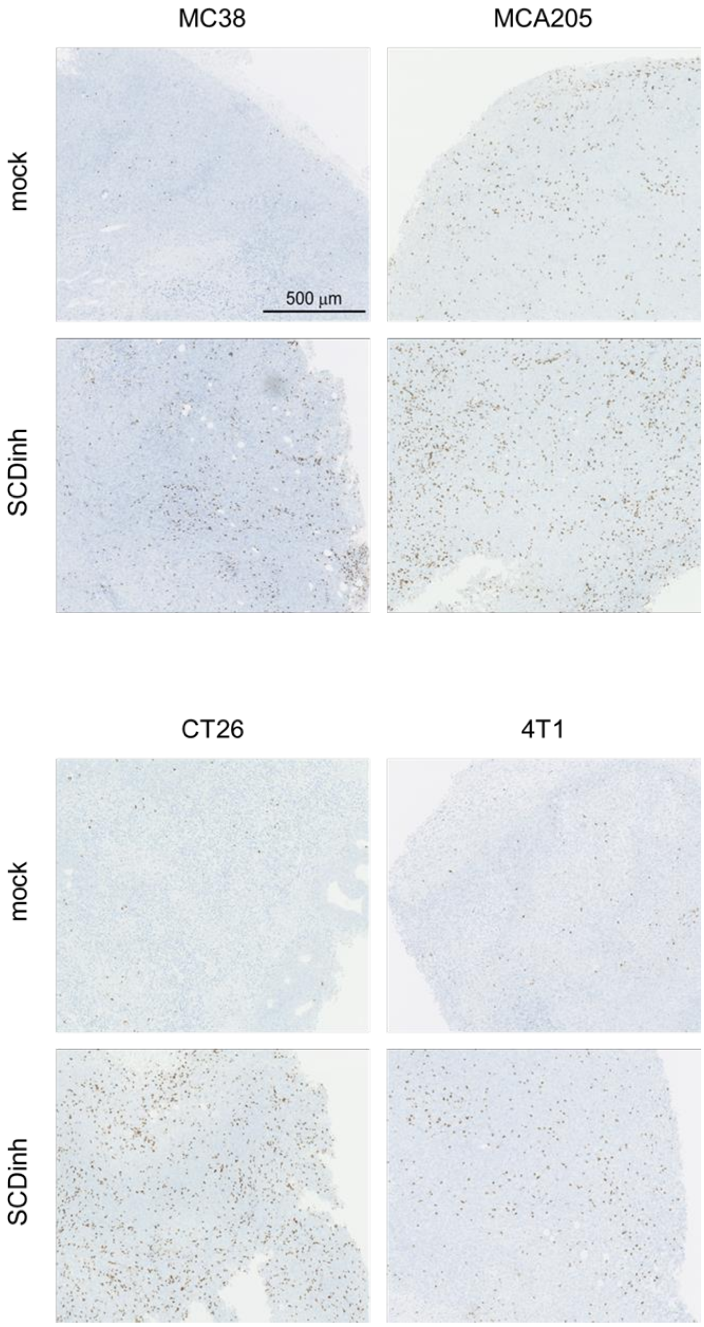

187 **Supplementary Figure 2. Inhibition of SCD1 enhances the infiltration of CD8$^+$ T cells.**

188 Large image of the IHC of tumor-infiltrated CD8$^+$ T cells shown in Figure 2A.

189

Katoh Y, *et al. J Immunother Cancer* 2022; 10:e004616. doi: 10.1136/jitc-2022-004616

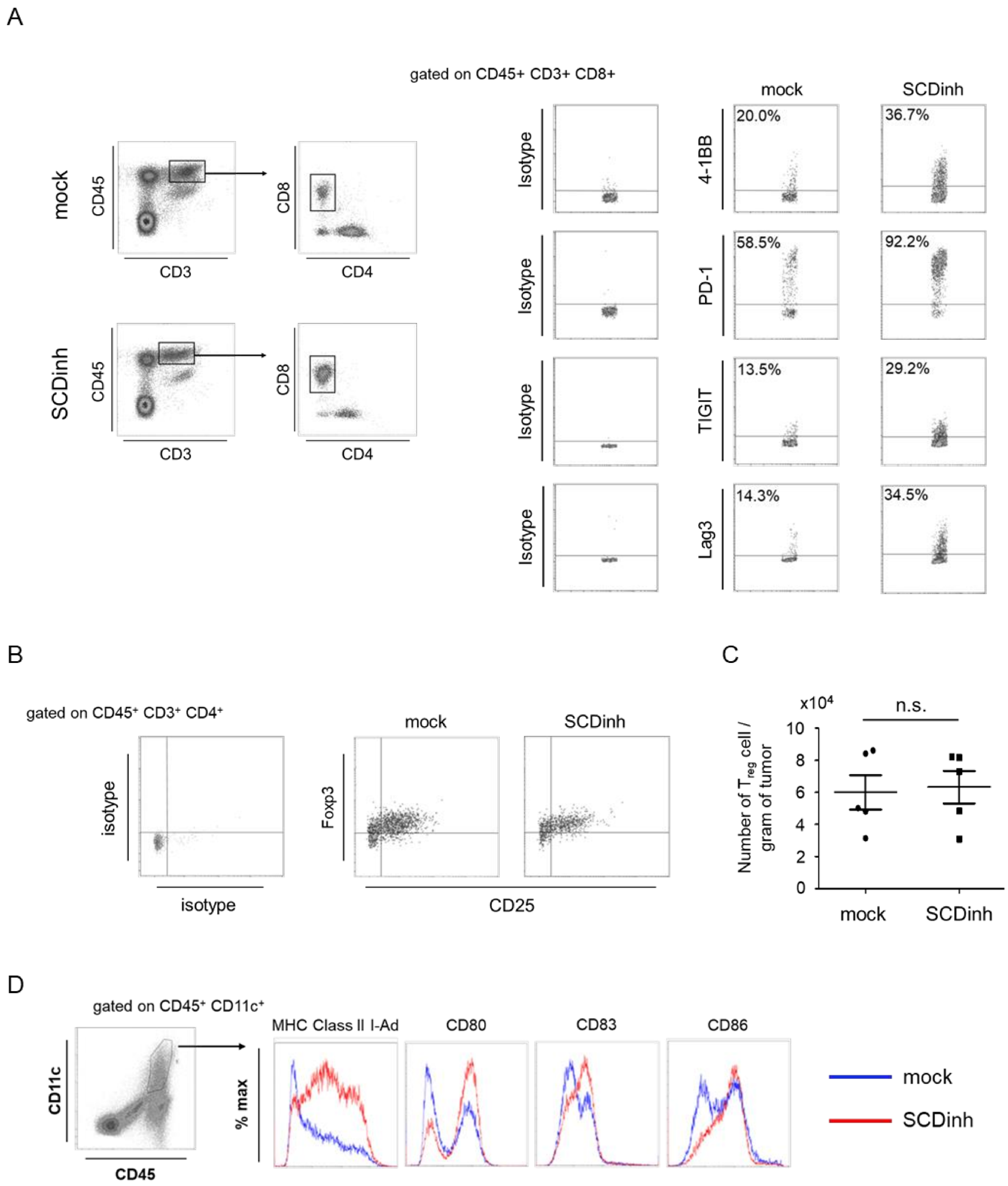

190 **Supplementary Figure 3. Phenotypic analysis of various tumor-infiltrating immune cells.**

191 **(A-C)** C57BL/6 mice bearing MC38 tumors were treated with a SCD inhibitor (SCDinh) or

with vehicle (mock). **(A)** Representative flow cytometry plots showing tumor-infiltrating CD8$^+$ T cells (left panel) and the expression of 4-1BB, PD-1, TIGIT, and Lag3 on them (right panel). **(B, C)** Representative flow cytometry plots for CD25 and FOXP3 staining of CD4$^+$ T cells **(B)** and absolute number of Treg cells in each mouse **(C)**. **(D)** Balb/c mice bearing CT26 tumors were treated with a SCD inhibitor (SCDinh) or with vehicle (mock). Data showing MHC Class II I-Ad, CD80, CD83 and CD86 expression gated on CD45$^+$ CD11c$^+$ cells in tumors.

Katoh Y, *et al. J Immunother Cancer* 2022; 10:e004616. doi: 10.1136/jitc-2022-004616

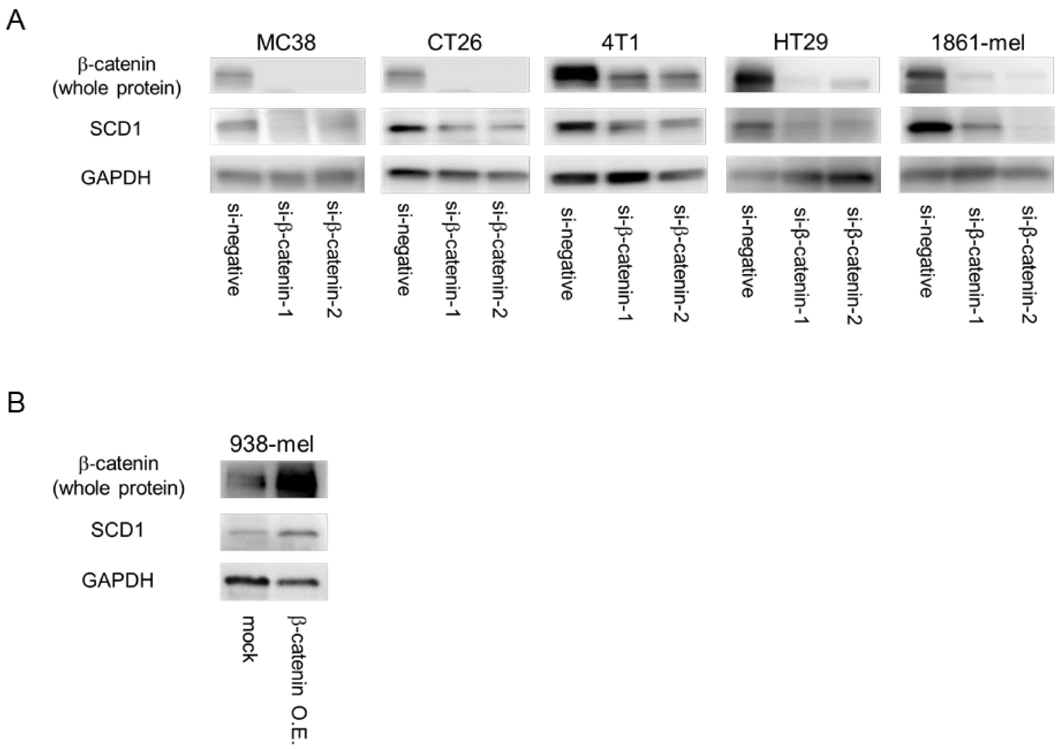

Supplementary Figure 4. Expression of SCD1 following knockdown or over-expression of β-catenin. **(A)** Mouse cancer cells (MC38, CT26 and 4T1) and human cancer cells (HT29 and 1861mel) were transfected with small interfering RNA (siRNA)-β-catenin or with siRNA-control (si-negative). Protein levels were measured by western blot 48 h after transfection. **(B)** SCD1 protein expression in human melanoma cells (938mel) that over-expressed β-catenin. GAPDH was used as a control. O.E., over-expressing.

Katoh Y, *et al. J Immunother Cancer* 2022; 10:e004616. doi: 10.1136/jitc-2022-004616

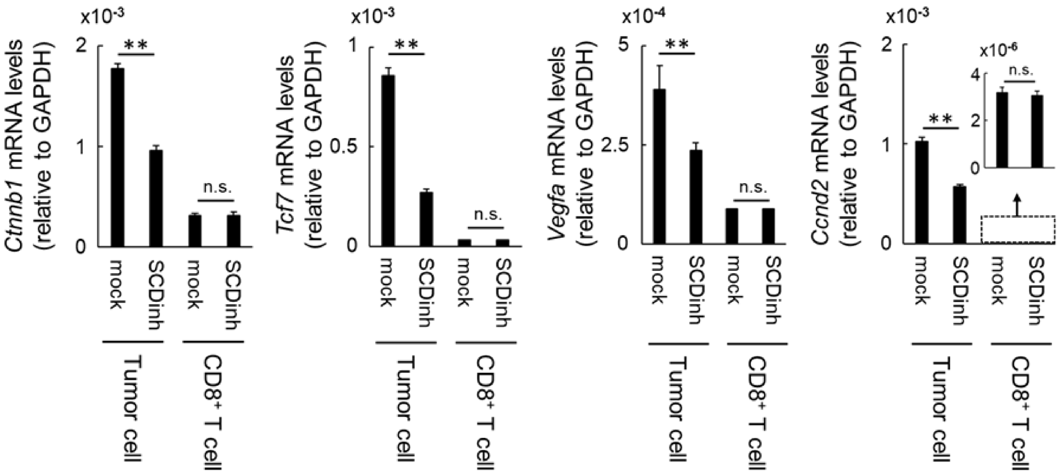

Supplementary Figure 5. Expression of β-catenin related gene in tumor cell and CD8$^+$ T cell. C57BL/6 mice bearing DsRed-MC38 tumors were treated with SCDinh or with vehicle (mock) after which DsRed$^+$ tumor cells and CD8$^+$ T cells were isolated at day 20. Total RNA was extracted and Ctnnb1, Tcf7, Vegfa and Ccnd2 gene expression was analyzed by real-time RT-PCR. Data are expressed as means ± SD (n=3). **P<0.01.

Katoh Y, *et al. J Immunother Cancer* 2022; 10:e004616. doi: 10.1136/jitc-2022-004616

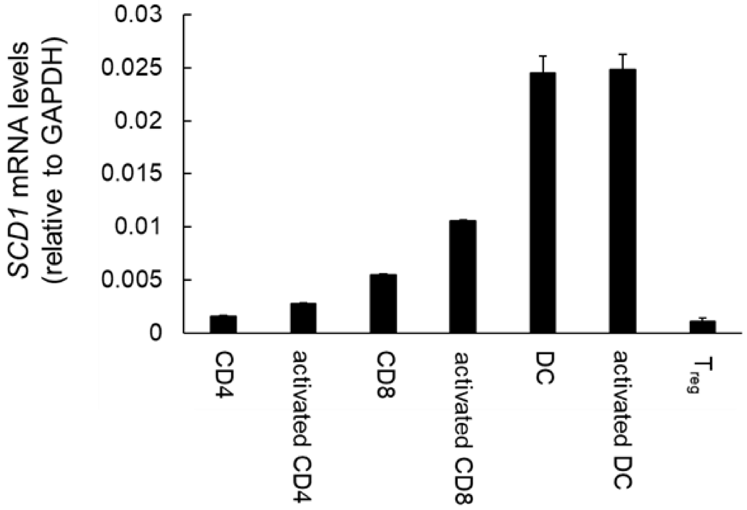

Supplementary Figure 6. Gene expression levels of SCD1 in various types of immune cells.

Human CD4$^+$ T cells and CD8$^+$ T cells were isolated from human PBMCs using MACS and were subsequently activated with an anti-CD3 monoclonal antibody, with an anti-CD28 monoclonal antibody and with interleukin-2. Human Treg cells were isolated from PBMCs using MACS. Human DCs were differentiated from CD14$^+$ PBMCs as described in the online supplemental Materials and Methods. Differentiated DCs were activated by LPS stimulation. Data are expressed as means ± SD (n=3).

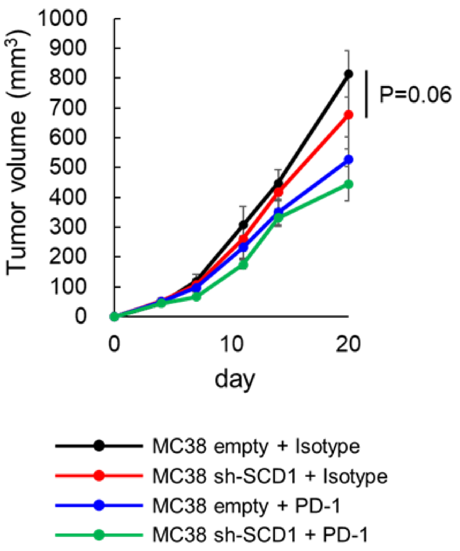

Supplementary Figure 7. Knockdown of SCD1 in cancer cells enhances the therapeutic effect of anti-PD-1 antibodies. C57BL/6 mice bearing vector control MC38 (MC38 empty) or SCD1-knockdown MC38 tumors (MC38 sh-SCD1) were treated with an anti-PD-1 (200 μg/mouse) or an isotype-matched antibody.

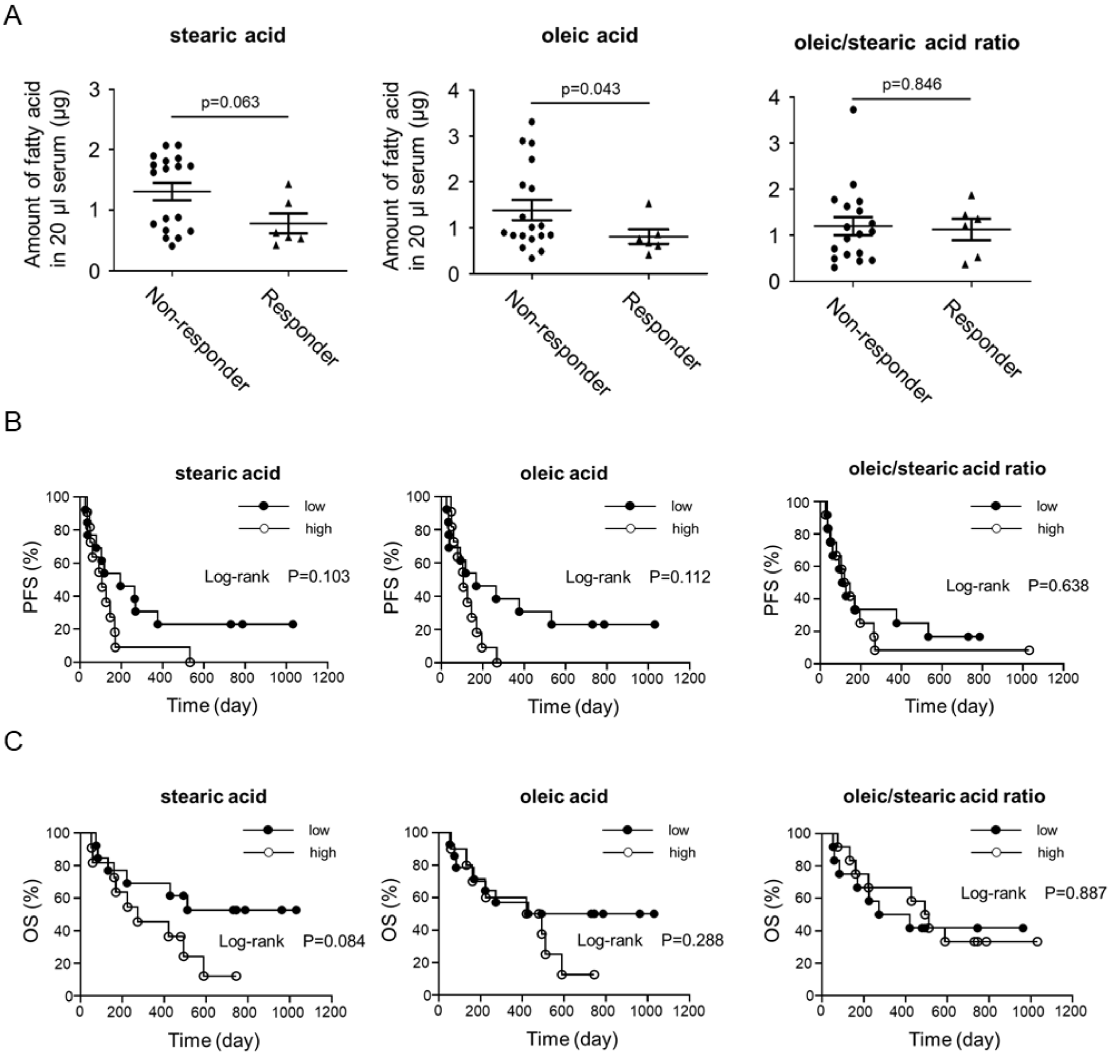

Supplementary Figure 8. Correlation of serum stearic and oleic acid levels and their ratio with clinical outcomes. (A) Pretreatment serum levels of stearic and oleic acid and ratio of oleic/stearic acid from NSCLC patients who subsequently did (Responder, n=6) or did not (Non-responder, n=18) respond to anti-PD-1 antibody treatment. **(B, C)** Kaplan–Meier analyses of NSCLC patients before anti-PD-1 antibody treatment. Kaplan–Meier curve of high (n=12) and low (n=12) stearic acid, oleic acid and oleic/stearic acid ratio in the serum of PFS patients

Katoh Y, *et al. J Immunother Cancer* 2022; 10:e004616. doi: 10.1136/jitc-2022-004616

(B) (HR 0.51, 95% CI 0.21 to 1.24; HR 0.53, 95% CI 0.22 to 1.28; HR 0.81, 95% CI 0.31 to 2.12, respectively) or OS patients **(C)** (HR 0.42, 95% CI 0.15 to 1.18; HR 0.58, 95% CI 0.22 to 1.55; HR 0.93, 95% CI 0.34 to 2.57, respectively). The threshold between high and low was median (stearic acid; 1.00, oleic acid; 0.85, oleic/stearic acid ratio; 1.13).

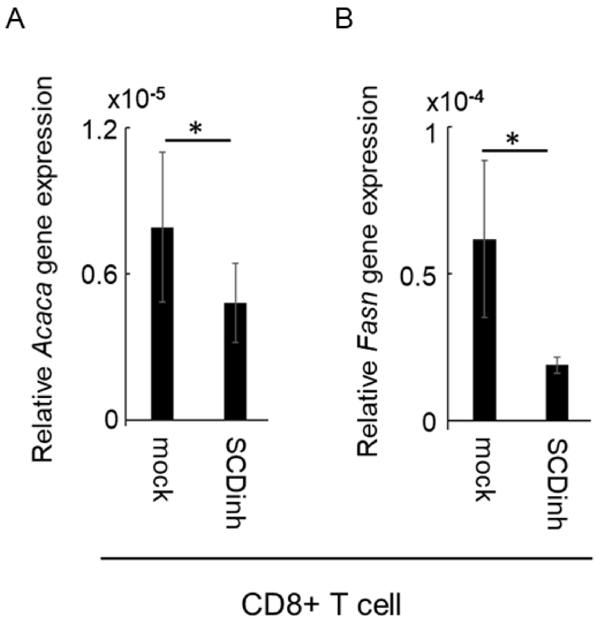

Supplementary Figure 9. Effect of a SCD1 inhibitor on lipogenic enzymes in CD8$^+$ T cells.

C57BL/6 mice bearing DsRed-MC38 tumors were treated with a SCD inhibitor (SCDinh) or with vehicle (mock). CD8$^+$ T cells were isolated from the bulk tumor at day 20 using a cell sorter. Acaca **(A)** and Fasn **(B)** gene expression levels were analyzed by real-time RT-PCR. Data are expressed as means ± SD (n=3). *P<0.05, **P<0.01.

Supplementary Tables

		Forward	Reverse
mouse	sXbp1	gctgagtccgcagcaggt	cagggtccaacttgtccagaat
	uXbp1	cagactacgtgcacctctgc	cagggtccaacttgtccagaat
	Scd1	acttaccaaagaccccaggc	accctgcattaacccccttc

Supplementary Table 1. Primer's list for qPCR (SYBR).

Katoh Y, *et al. J Immunother Cancer* 2022; 10:e004616. doi: 10.1136/jitc-2022-004616

mouse	si-negative	Sense	siRNA Universal Negative Control (sigma)
		Antisense	siRNA Universal Negative Control (sigma)
	si-β-catenin	Sense	CACUUGCAAUAAUUACAAAtt
		Antisense	UUUGUAAUUAUUGCAAGUGag
	si-β-catenin	Sense	GACUCAAUACCAUUCCAUUtt
		Antisense	AAUGGAAUGGUAUUGAGUCct
	si-Scd1 1	Sense	GGGAUUUUCUACUACAUGAtt
		Antisense	UCAUGUAGUAGAAAAUCCCga
	si-Scd1 2	Sense	CCUUCUUGCGAUACACUCUtt
		Antisense	AGAGUGUAUCGCAAGAAGGtg
	si-Atf3 1	Sense	GGAUUUUGCUAACCUGACAtt
		Antisense	UGUCAGGUUAGCAAAAUCCtc
	si-Atf3 2	Sense	AGAGGAACCUCUUUAUCCAtt
		Antisense	UGGAUAAAGAGGUUCCUCUcg
human	si-negative (for β-catenin)	Sense	ACAAUCGGUUAUAGUUAGGAGACAG
		Antisense	UGUCUCCUAACUAUAACCGAUUGU
	si-negative (for SCD1 and ATF3)	Sense	siRNA Universal Negative Control (sigma)
		Antisense	siRNA Universal Negative Control (sigma)
	si-β-catenin 1	Sense	ACAAGUAGCUGAUAUUGAUGGACAG
		Antisense	CUGUCCAUCAAUAUCAGCUACUUGU
	si-β-catenin 2	Sense	CAGUUAUGGUCCAUCAGCUUUCUAA
		Antisense	UUAGAAAGCUGAUGGACCAUAACUG
	si-SCD1 1	Sense	CCAGAGGAGGUACUACAAAtt
		Antisense	UUUGUAGUACCUCCUCUGGaa
	si-SCD1 2	Sense	CCCUGUAUGGGAUCACUUUtt
		Antisense	AAAGUGAUCCCAUACAGGGct
	si-ATF3 1	Sense	GCAAAGUGCCGAAACAAGAtt
		Antisense	UCUUGUUUCGGCACUUUGCag
	si-ATF3 2	Sense	CUGGGUCACUGGUGUUUGAtt
		Antisense	UCAAACACCAGUGACCCAGga

Supplementary Table 2. List of si-RNAs for mouse and human target gene.
