# Supplementary material for: Inhibition of stearoyl-CoA desaturase 1 (SCD1) enhances the antitumor T cell response through regulating β-catenin signaling in cancer cells and ER stress in T cells and synergizes with anti-PD-1 antibody
Source: J Immunother Cancer. 2022 Jul 6;10(7):e004616. doi: 10.1136/jitc-2022-004616 (PMC9260842; doi:10.1136/jitc-2022-004616)
Supplement: Supplementary data [file jitc-2022-004616supp003.pdf]

Inhibition of stearoyl-CoA desaturase 1 (SCD1) enhances the anti-tumor T cell response through regulating β-catenin signaling in cancer cells and ER stress in T cells and synergize with anti-PD-1 antibody

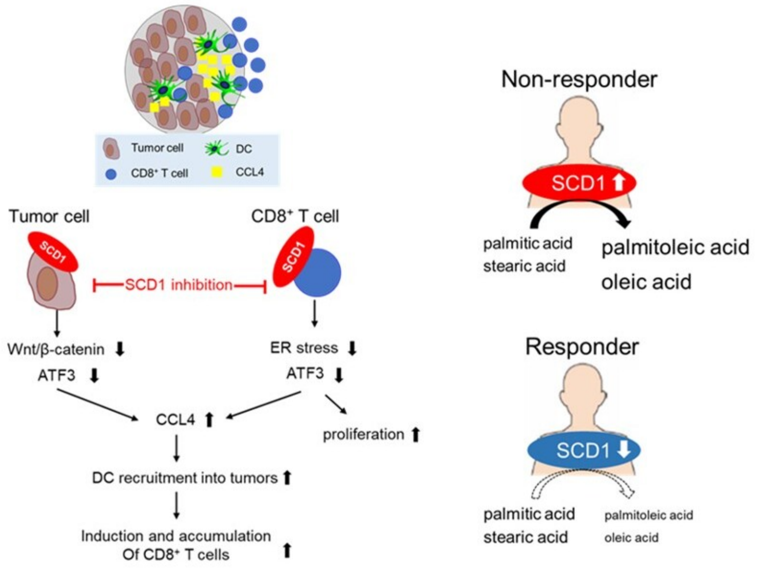

Authors

Yuki Katoh, Tomonori Yaguchi, Akiko Kubo, Takashi Iwata, Kenji Morii, Daiki Kato, Shigeki Ohta, Ryosuke Satomi, Yasuhiro Yamamoto, Yoshitaka Oyamada, Kota Ouchi, Shin Takahashi, Chikashi Ishioka, Ryo Matoba, Makoto Suematsu and Yutaka Kawakami.

In Brief

Inhibition of SCD1 in cancer cells via suppressing β-catenin signaling and in effector T cells via reducing ER stress enhances anti-tumor T cells through CCL4 recruited dendritic cells, and synergizes with anti-PD-1 antibody. Patients with high serum SCD1 related fatty acids were non-responders to anti-PD-1 antibody therapy.
