# Supplementary figures and images for: Inhibition of stearoyl-CoA desaturase 1 (SCD1) enhances the antitumor T cell response through regulating β-catenin signaling in cancer cells and ER stress in T cells and synergizes with anti-PD-1 antibody
Source: J Immunother Cancer. 2022 Jul 6;10(7):e004616. doi: 10.1136/jitc-2022-004616 (PMC9260842; doi:10.1136/jitc-2022-004616)

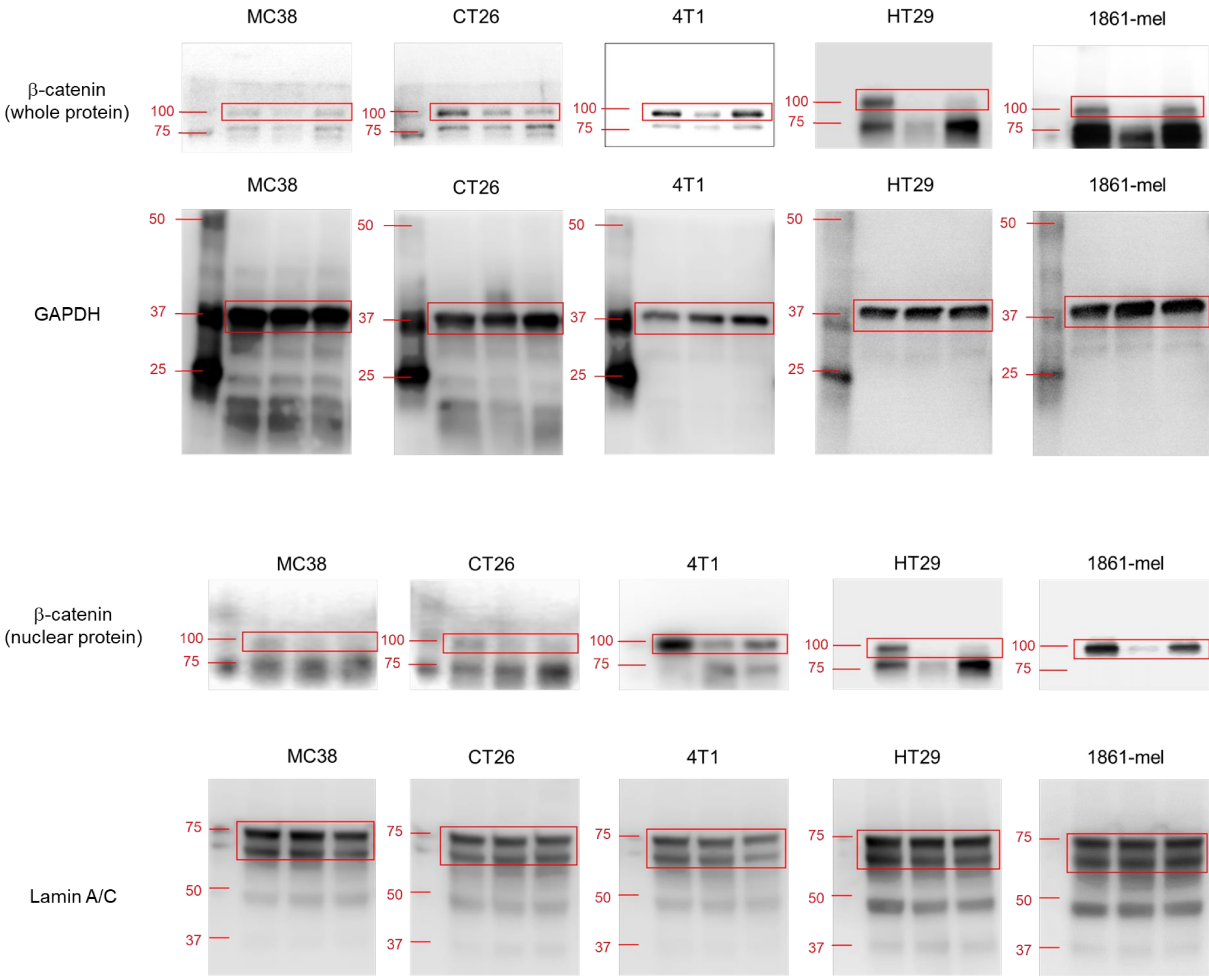

Source data 1 (related to figure 3E)

Supplement: Supplementary data [file jitc-2022-004616supp004.pdf]

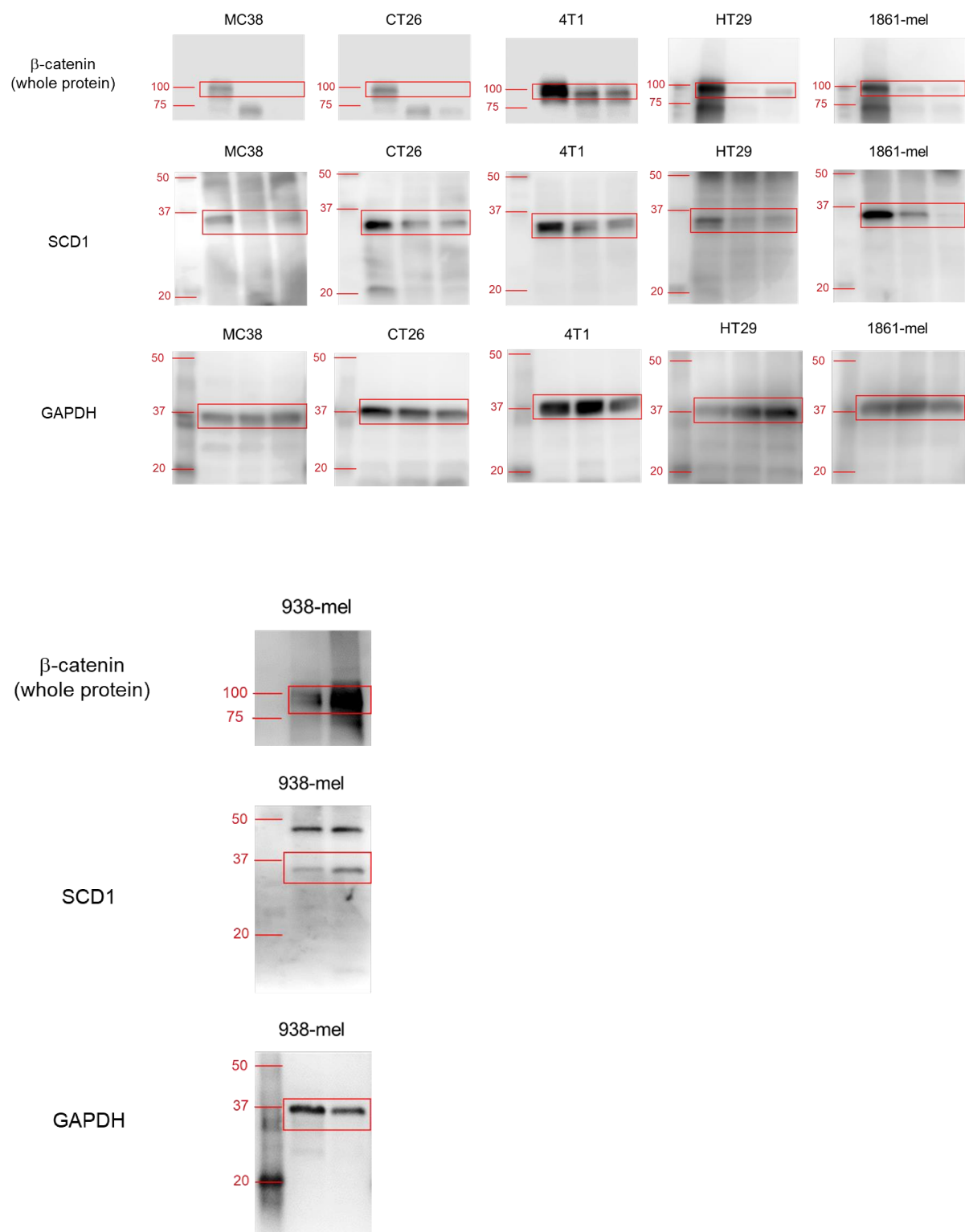

Source data 2 (related to supplemental figure 5)

Supplement: Supplementary data [file jitc-2022-004616supp005.pdf]
